# Selective Delivery to Cardiac Muscle Cells Using Cell-Specific Aptamers

**DOI:** 10.3390/ph16091264

**Published:** 2023-09-06

**Authors:** Styliana Philippou, Nikolaos P. Mastroyiannopoulos, Marios Tomazou, Anastasios Oulas, Matthew Ackers-Johnson, Roger S. Foo, George M. Spyrou, Leonidas A. Phylactou

**Affiliations:** 1 Department of Molecular Genetics, Function & Therapy, The Cyprus Institute of Neurology and Genetics, Nicosia 2371, Cyprus; 2 Department of Bioinformatics, The Cyprus Institute of Neurology and Genetics, Nicosia 2371, Cyprus; 3 Cardiovascular Research Institute, Centre for Translational Medicine, National University of Singapore, Singapore 117599, Singapore

**Keywords:** aptamer, SELEX, cardiomyocytes, delivery, heart, muscular dystrophy

## Abstract

In vivo SELEX is an advanced adaptation of Systematic Evolution of Ligands by Exponential Enrichment (SELEX) that allows the development of aptamers capable of recognizing targets directly within their natural microenvironment. While this methodology ensures a higher translation potential for the selected aptamer, it does not select for aptamers that recognize specific cell types within a tissue. Such aptamers could potentially improve the development of drugs for several diseases, including neuromuscular disorders, by targeting solely the proteins involved in their pathogenesis. Here, we describe our attempt to utilize in vivo SELEX with a modification in the methodology that drives the selection of intravenously injected aptamers towards a specific cell type of interest. Our data suggest that the incorporation of a cell enrichment step can direct the in vivo localization of RNA aptamers into cardiomyocytes, the cardiac muscle cells, more readily over other cardiac cells. Given the crucial role of cardiomyocytes in the disease pathology in DMD cardiomyopathy and therapy, these aptamers hold great potential as drug delivery vehicles with cardiomyocyte selectivity.

## 1. Introduction

Aptamers are a class of synthetic DNA or RNA molecules that can act as affinity probes or molecular recognition elements for a variety of applications from biosensing to therapeutics and most importantly, as ligands for targeted drug delivery. Aptamers are single stranded oligonucleotides which fold into complex three-dimensional structures capable of binding to a wide range of target molecules with high affinity and specificity [1]. Given the high precision in their binding, aptamers can differentiate between molecules that differ by as little as one functional group. Aptamers bind their cognate targets in a manner similar to how antibodies recognize their antigen counterparts, using a combination of van der Waals forces, hydrogen bonding, electrostatic interactions, and shape complementarity [2]. Therefore, aptamers are often called “chemical antibodies”. Compared to antibodies, aptamers have several advantages primarily due to their smaller size that makes tissue penetration easier, even for difficult targets such as therapeutic targeting of the blood–brain barrier [3]. Given their nucleic acid characteristics, aptamers are not directly recognized by the immune system, and thus raise low to no immune response and/or low toxicity in therapeutic application [1]. Additionally, aptamers are produced via chemical synthesis that makes them thermally more stable than antibodies and thus can be stored and transported even at room temperature. Furthermore, they can be produced on a large scale with minimal batch-to-batch variations and in a short time, whereas their ability to be amenable to chemical modifications enhances their resistance against nucleases in vivo. The 2′-Fluoro (2′-F) and 2′-O-methyl (2′-OMe) are two common 2′-substitute modifications on the ribose ring, frequently utilized during aptamer development to increase nuclease resistance and aptamer binding affinity [4,5]. All these characteristics suggest superior clinical applicability and suitability of aptamers over antibodies.

Aptamers are identified via Systematic Evolution of Ligands by Exponential Enrichment (SELEX) from a large pool of random sequences [3,6,7]. The core of the process involves three basic steps: (i) incubation of a pool of random sequences with the target, (ii) partitioning of the bound from the non-bound molecules, and (iii) recovery and amplification of the bound molecules. The process is repeated and, following several rounds, enriched sequences emerge as aptamer candidates with higher binding affinity and specificity for the target. Substantial characterization then identifies the best-performing aptamer among the emerging candidates. Variations of the SELEX platform have given the capability to bind numerous targets including organic compounds, nucleotides, proteins and even whole cells and tissues in living organisms. Cell internalization SELEX is one of the most applied variations. It identifies aptamers that internalize and can potentially deliver agents intracellularly, thus acting as targeting ligands [1]. This is done by developing the aptamers against either known or unknown membrane receptors directly within their native environment, allowing a straightforward enrichment of cell-specific aptamers [7]. Numerous therapeutic reagents have, thus far, been successfully delivered using cell-specific aptamers including small interfering RNAs (siRNA), microRNAs, nanoparticles, chemotherapeutics and toxins [8,9,10,11,12].

A more recent trend in aptamer utilization as targeted ligands is their conjugation to lipid drug carriers, such as liposomes and micelles or polymer-based nanocapsules, with drugs being place in the core cavity of these molecules and thus, providing protection against rapid degradation, controlled drug release, delivery of higher drug concentrations to targeted sites and subsequent reduced toxicity to non-targeted organs [13]. For instance, conjugation of the nucleolin aptamer AS1411 on liposomes loaded with the anticancer drug 5-fluorouracil induced a more pronounced cytotoxic effect to TE 354.T cells, compared to the non-functionalized liposomes, demonstrating the efficiency of liposomes functionalized with AS1411 aptamer as an alternative for the treatment of basal cell carcinoma [14]. The same aptamer was conjugated to micelles to improve the anti-cancer efficacy of doxorubicin and miR-519c for hepatocellular carcinoma [15]. The micelles demonstrated cellular uptake and tumor penetration ability driven by the AS1411 aptamer whereas the co-delivery of doxorubicin and miR-519c resulted in efficient tumor growth inhibition. Successful co-delivery of another anticancer drug, docetaxel, and the insulin growth factor receptor 1 siRNA was demonstrated by the conjugation of the anti-Mucin 1 aptamer to chitosan nanoparticles [16]. In this example, the cellular uptake of the nanoparticles was enhanced whereas delivery of docetaxel along the siRNA resulted in a decrease in cell viability of SKBR3 metastatic breast cancer cells and genes expressions involved in the progression of the tumor and metastasis in vitro. Therefore, aptamer functionalization on the surface of nanocarriers may offer a higher therapeutic opportunity to nanocarriers via the active targeting of cells.

The more recent in vivo SELEX generates tissue/organ-targeted aptamers directly within living animals [3,17,18,19,20]. The nuclease-resistant aptamer library is administered intravenously (IV), followed by harvesting of the organ of interest to extract the bound sequences. The use of IV administration offers a great advantage, as the aptamer pools are exposed to all organs during circulation and are given the choice of target [18]. Furthermore, the pharmacokinetics and tissue distribution profile are taken into account during selection, thus limiting extensive post-SELEX optimizations and leading to the recovery of aptamers with a potentially higher in vivo specificity. This methodology has already been applied for the development of functional aptamers against healthy and diseased tissue microenvironments, such as the brain and various tumors [3,17,18,19,20]. Nevertheless, none of these aptamers can selectively bind onto specific cell types within tissues.

**Figure 1:**
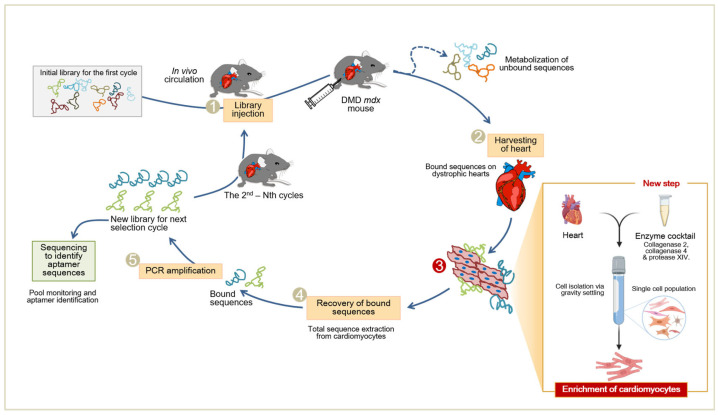
In vivo Cell SELEX platform for cardiomyocytes (CMs). After systemic administration and circulation of the 2’F-Py RNA library in the DMD*^mdx^* mouse (step 1), dystrophic hearts are harvested for subsequent isolation of CMs (step 2). In this way only sequences bound on CMs are extracted (step 3—new). The recovered 2’F-Py RNA sequences are re-amplified to make a new 2’F-Py RNA library for the next selection round (step 4). This procedure is repeated until the library becomes enriched with 2’F-Py RNA sequences that present CM selectivity (The figure was recreated from Reference [21], Copyright © 2016, Springer Nature Limited). See also Figure S1 and Table S1.

In this study, we investigate the incorporation of a cell-enrichment step prior to the recovery of intravenously injected aptamers as a means to enhance the selectivity of the aptamers towards the cardiomyocyte portion of the heart. Using the in vivo Cell SELEX adaptation, cardiomyocytes (CMs) were identified with more ease by the enriched aptamer pools as well as the selected 10,478 2’F-Py RNA aptamer, despite the complex microenvironment of the heart (Figure 1). More importantly, as the selection was performed against dystrophic hearts, this work opens the possibility for aptamer-based in vivo therapeutic targeting of muscular-dystrophy-related cardiomyopathies.

## 2. Results

### 2.1. In Vivo SELEX for Cardiomyocyte Selection

**Figure 2:**
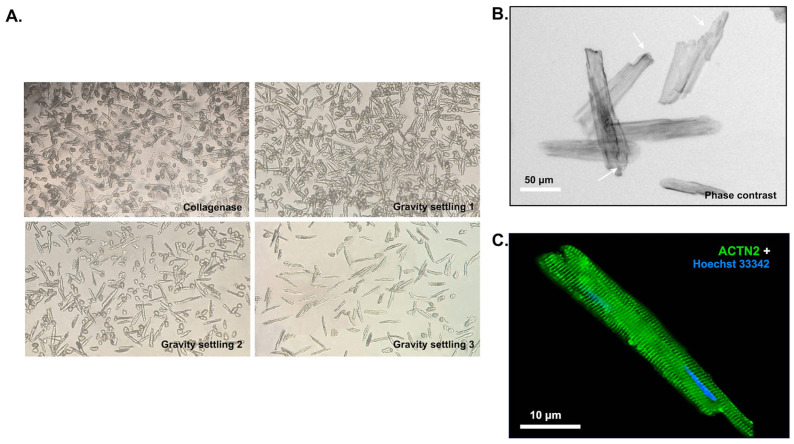
Morphology of isolated ventricular CMs. (**A**). Representative images of ventricular CMs during isolation using a light microscope (×5 magnification) and gravity settling. Rod-shaped cells denote CMs; circular cells denote non-CMs and dead cells; dark spots denote debris and undigested tissue. (**B**). Isolated CMs exhibit a characteristic rod-shaped morphology with “stepped” ends (arrows) and clear cross-striations (scale bar = 50 μm). (**C**). Antibody specificity. Representative fluorescent microscopy image of a single CM isolated from *mdx* mice and stained with sarcomeric-α-actinin antibody (α-ACTN2, green), and Hoechst 33342 nuclear counterstain (blue), after isolation and a 48-h culture. α-ACTN2 localizes in the Z-discs of sarcomeres resulting in the characteristic cross-striated appearance of CMs (scale bar = 10 μm).

To further enhance the sensitivity of in vivo SELEX, a cell-enrichment step was incorporated in the selection of aptamers as shown in Figure 1. As we were interested in the development of RNA aptamers for DMD-related cardiomyopathies, the cell enrichment was driven towards dystrophic CMs using a methodology previously described [22]. The CM isolation technique for DMD*^mdx^* mice was sufficiently optimized before being incorporated into the in vivo selection rounds, by monitoring the purity, viability and morphology of recovered CMs. Purified CMs were separated from the non-CM (single cell) heart population using sequential gravity settling as shown in Figure 2A. To further assess the output of the enrichment method, cells were counted using an automated cell counter. As demonstrated in Table 1, the CM yield and viability from DMD*^mdx^* mice were similar to previously reported isolations from healthy mice [22]. Furthermore, the isolated CMs maintained the distinctive rod-shaped morphology and “stepped ends” of native CMs whereas immunostaining with sarcomeric α-actinin (α-ACTN2) that binds onto the Z-discs of sarcomeres further showed maintenance of intact cellular structures in CMs (Figure 2B, arrows and Figure 2C).

To develop aptamers as therapeutic targeting ligands for cardiomyopathies, the initial pool of aptamers was intravenously injected into DMD*^mdx^* mice, a commonly used mouse model for studying Duchenne muscular dystrophy (DMD) [23]. A 77-base-long library of 2′-fluoropyrimidine (2′F-Py RNA) nuclease-resistant RNA was used as the starting (initial) pool and a total of seven selection rounds were performed (Figure S1A,B). For each round, the aptamer library was injected into three DMD*^mdx^* mice, hereafter referred to as *mdx* mice, and allowed to circulate for 2 h before harvesting the heart for CM enrichment. Owing to the already stringent nature of the in vivo Cell SELEX, the selection conditions were mainly unaltered (Figure S1C). To monitor the degree of enrichment during selection, the recovered RNA pools from all seven rounds were analyzed using conventional PCR (Figure S1C, bottom). The earlier the PCR product band was detected, the more enriched the pools were in aptamer sequences. Assessing the binding/internalization ability of the pools using quantitative real-time PCR (RT-qPCR) further showed the presence of strong binders/internalizers in Rounds 6 and 7, with a 5-fold difference from Round 1 binders (Figure S1D).

### 2.2. Enriched Aptamer Pool Selectivity for Cardiomyocytes

**Figure 3:**
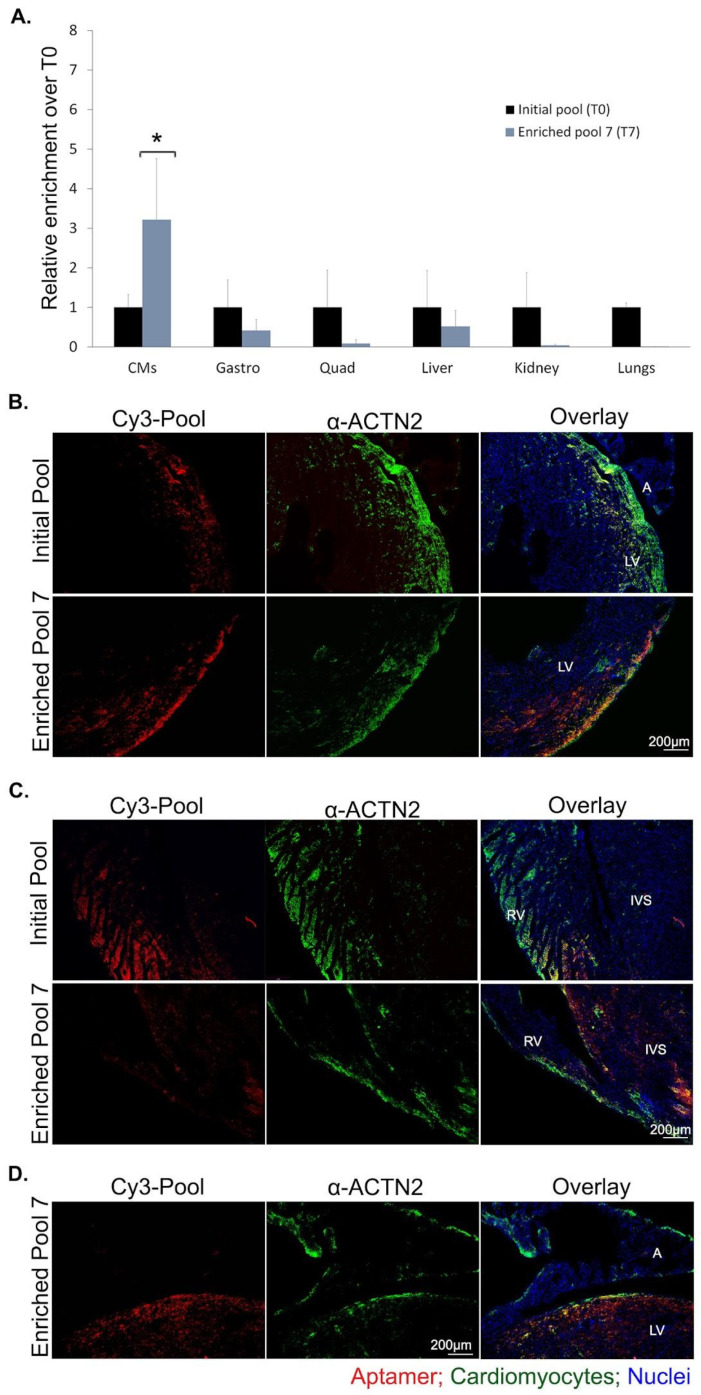
Enriched aptamer pool localization in ventricular CMs. (**A**). Quantitation of the initial pool (T0) or enriched pool #7 (T7) enrichment, following IV injections in *mdx* mice and isolation of selected organs 2 h after the injection (n = 3). Quantities were calculated using the absolute quantification (RT-qPCR) standard curve method, normalized to tissue mass (ng/mg of tissue) and then expressed as fold change relative to the background binding detected with the initial pool (T0) used for selection (mean + SD; * *p* < 0.05). CMs, cardiomyocytes; gastro, gastrocnemius; quad, quadriceps. (**B**). IV injection in *mdx* mice (n = 3 per aptamer) of 5 nmol (~124 μg) Cy3-labeled initial 2′F-Py RNA pool or enriched 2′F-Py RNA pool #7 (red) followed by collection of tissues 2 h after injection. Sections were immunostained with α-ACTN2 for CMs identification (green) and counterstained with Hoechst 33342 nuclear stain (blue). Representative images of left ventricles (LV), where most CMs localize, captured using the same laser intensities and acquisition time. Areas of colocalization are shown in yellow (initial pool shows an area of localization in the upper left ventricle whereas for the enriched pool localization was mostly detected in the lower left ventricle (scale bar = 200 µm). (**C**). Representative fluorescent microscopy images of right ventricle (RV) and interventricular space (IVS) heart sections from experiment in (**A**) for comparison of localization. (**D**). Representative fluorescent microscopy images depicting the selective localization of the enriched aptamer pool in the left ventricular CMs and not the left atrial CMs. See also Figures S2 and S3 and Statistical Analysis section in the Supplementary Materials.

To further assess the level of selective binding in CMs, we evaluated the localization of the enriched pool 7 in various tissues following intravenous administration. The analysis focused on tissues that could potentially have similar surface receptors with CMs, such as skeletal muscles, as well as highly perfused organs (i.e., liver, kidney and lungs). The level of localization was quantitated using absolute RT-qPCR (the dynamic linear range of this assay is given in Figure S2). As shown in Figure 3A, the enriched pool 7 (T7) presents an affinity for CMs that is almost three times more than the initial pool (T0). Furthermore, the non-targeted organs show low T7 uptake that is below the background T0 uptake. As the protocol used for CM isolation favors left ventricular CMs, we next sought to evaluate whether the enriched pool 7 presents with selective ventricular CM localization. Longitudinal heart sections obtained from *mdx* mice injected with the initial library showed little and random localization in CMs (indicated by few areas of colocalization), whereas the enriched pool 7 had a similar (aptamer) staining pattern with left ventricular CMs (Figure 3B). On the contrary, images obtained from portions of the right ventricle and atria show little to no difference among the initial and enriched pool 7, thus confirming the selective enrichment of left ventricular CMs as the protocol implies (Figure 3C,D). Additionally, administration of the pools in healthy mice showed a similar result, suggesting potential aptamer binding onto a receptor(s) that is present in both states (Figure S3).

### 2.3. Sequencing Analysis of Aptamer Enrichment

**Figure 4:**
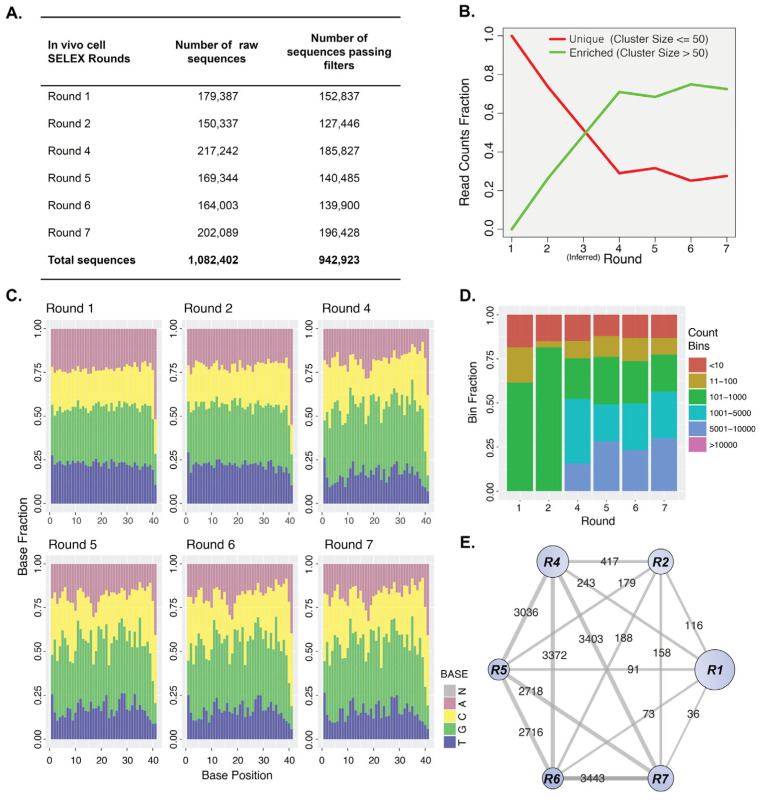
NGS data analysis of aptamer enrichment for CMs. (**A**). Summary of the NGS data successfully sequenced and analyzed. (**B**). Analysis of the unique and enriched sequences over the course of the selection. Clusters with 50 sequences or fewer are categorized as unique clusters (i.e., lost in subsequent rounds) whereas clusters with more than 50 sequences are classified as enriched clusters (i.e., clusters with sequences being enriched/favored in selection). Read count fraction denotes the proportion of the read counts (sequences) being enriched or lost over the total population of sequences, where the total population equals 1. (**C**). Nucleotide distribution over the 40 positions of the central random region of the recovered 2’F-Py RNA libraries. Pink: dA, green: dG, dark yellow: dC, blue: dT and grey: N, unknown nucleotides. (**D**). Categorization of clusters into 6 bins (groups) according to the number of read counts. Bin fraction denotes the proportion of each bin of the total population of sequences (=1). (**E**). Common sequences network between rounds. Node sizes (blue) denote the decrease in the pool heterogeneity. Edges (grey) and corresponding labels show common aptamer clusters between SELEX rounds. See also Table S2, Figures S4 and S5.

In order to prepare the libraries for next generation sequencing (NGS), adapter sequences and unique indices were added onto each pool as shown in Figure S4. To distinguish the origin of each aptamer sequence (i.e., the selection round to which they belong) different index combinations were used as shown in Table S2, whereas the overall success of the sequencing run was evaluated by determining the number of reads passing a filter (Figure 4A). A custom bioinformatics workflow (see Section 4) was built to denote the level of enrichment, relationship of sequences between rounds and number of reads per round as well as the sequence of highly ranked and frequently occurring clusters in the selection. As demonstrated in Figure 4B, the selection converged rapidly between Rounds 4–6. This could be the result of the highly stringent selection applied by the intravenous circulation of aptamers in the bloodstream. An increase in the number of enriched sequences with a simultaneous decrease in the number of unique sequences (<50 reads cluster) was observed, with a potential peak in enrichment between Rounds 4 and 7. The nucleotide distribution among the central 40-base random region verified the previous result (Figure 4C). This result further suggested a potential dominance of specific sequences in the enriched pools (a characteristic of an aptamer pool that is near its enrichment plateau phase). Additionally, sequence clusters of larger bin sizes (1001–5000 and 5001–10,000 read counts bins) were detected in Rounds 4–7 (Figure 4D), adding to the observation of dominant aptamer sequences in these rounds. To assess whether the aptamer sequences originated from previous rounds, a network of the rounds connected by their common sequences was created as demonstrated in Figure 4E. The presence of grey edges originating from and to all seven rounds indicates the existence of common sequences among them. The darker edges seen in Rounds 4 through 7 indicate larger absolute interactions between corresponding rounds, most probably due to similarities in the sequence diversity of these rounds and the increase in the abundance of strong aptamer binders. This result is further reinforced by the round-representation histograms and the drop in diversity index values presented in Figure S5.

### 2.4. Identification of Candidate Aptamer Clusters

**Figure 5:**
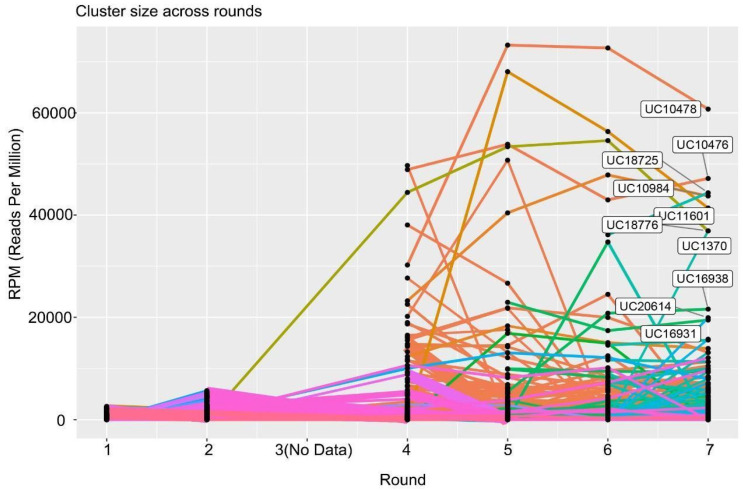
Aptamer cluster tracking across SELEX rounds. Aptamer sequences present in each round were grouped in clusters according to sequence similarities. Colors depict individual aptamer clusters that progress through rounds. The *Y*-axis shows the frequency of each cluster at each round in reads per million (normalization for sequence depth). The clusters with the highest number of reads at the final SELEX round are denoted by a unique cluster identification number (UC#). See also Appendix B and Table S3.

To identify possible aptamer candidates for subsequent individual assessments, NGS data were classified into clusters according to sequence similarities, with the most represented sequence being the seed sequence of each cluster (Figure 5). UC10478 was the most represented cluster with an almost 14,250 RPM difference from the next-best cluster UC10476 (see also Table S3 for frequencies). In addition, UC10478 was the lead cluster for the last three rounds suggesting potential specific-CM targeting. Next, the enrichment profile of the top 100 and then top 10 (Appendix B) clusters from Round 4 onwards was analyzed for frequency counts, sequence similarity, motifs and other patterns, revealing three additional clusters that could be individually characterized (UC10984, UC18725, and UC11601). Interestingly, for all these clusters, the seed sequence represented ≥83% of the population, suggesting high similarity of the enriched sequences within a corresponding cluster. To assess whether any of these 5 aptamer candidates shared conserved regions that could denote potential binding sites and direct future truncation studies, their primary sequences were aligned using Clustal Omega and their genetic relationship analyzed by constructing a phylogenetic tree. As demonstrated in Figure S6A, the sequences show little homology among them (asterisks), which contrasts with the phylogenetic tree result that shows UC10478 and UC10984 to be closely related. Furthermore, ex vivo staining of cryosections obtained from the heart, kidney and liver of *mdx* mice showed punctate red staining only for the first three aptamer sequences (Figure S6B). As an attempt to explain the staining pattern observed in the microscopy experiment, we generated and compared the secondary structure and helicity prediction of the lead aptamer candidate 10478 to that of the other four aptamer candidates, using the seed sequence of each cluster as input in NUPACK 4 software (Figure S7). The overall secondary structure of 10478 was mostly similar to 11601 whereas from the helicity predictions one region was mostly similar between 10478 and 10476 (purple rectangle).

### 2.5. Secondary NGS Analysis for Diversity Confirmation

Given the variable results obtained from the initial individual assessment of the aptamer candidates described in the previous section, we wondered whether the plateau enrichment was not at Round 7. Furthermore, most in vivo SELEX publications report ≥10 selection rounds to achieve pool enrichment [4,6,10,11,13,14]. For these reasons, two additional selection rounds were performed in a total of 6 *mdx* mice and then all nine rounds (Rounds 1–7 plus confirmatory Rounds 8 and 9) were subjected to a secondary NGS analysis (Figure S8). This (NGS) analysis was further complemented by the initial random pool (i.e., before being injected in mice) to further assess the origin of the enriched sequences (Figure S9). In general, the pool diversity is low and enrichment of additional sequences does not seem to occur in the confirmatory rounds. This is primarily evident from the analysis of the unique sequences across the selection rounds shown in Figure S9A. An enrichment level of ~80% is maintained throughout Rounds 4–9 whereas the diversification level falls below 20% at approximately the same time point in the selection. The nucleotide distributions analysis (Figure S9C) shows a gradual distortion in the nucleotides per position which further reinforces the aforementioned result.

Tracking the aptamer clusters across rounds showed the emergence of the same top clusters with a slight change in their ranking (Figure S10A). Aptamer candidate 10478 remained in the top position although its frequency increased in these last two confirmatory rounds. More specifically, the frequency of the selected clusters that were still present in Round 9 is as follows: UC10478, 300000 RPM; UC10984 and UC11601 ~37500 RPM and UC10476, ~32000 RPM. With more than 250000 RPM difference from the second-best cluster, stopping the selection at Round 7 did not affect the selection of UC10478 as the first aptamer candidate to be individually assessed for selective CM localization (Figure S10B).

### 2.6. 2′F-Py RNA Aptamer 10478 Shows Selective Localization in CMs

**Figure 6:**
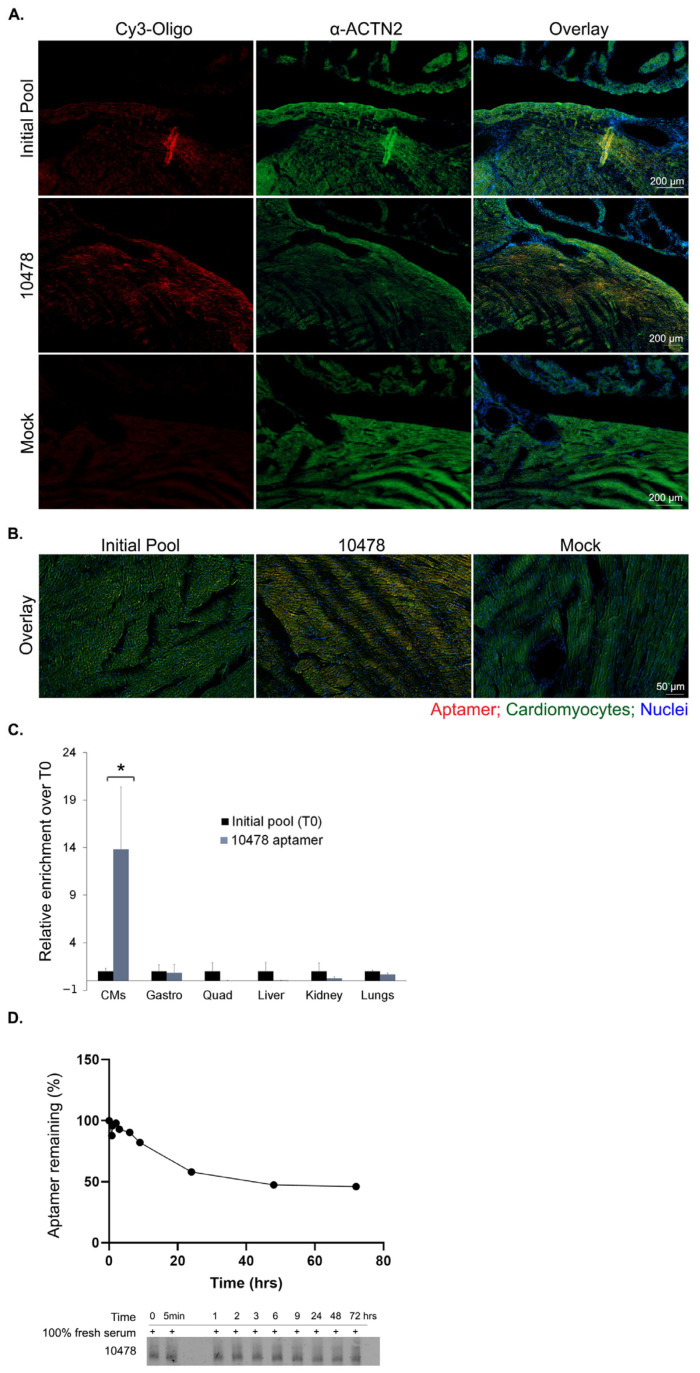
Selective localization aptamer 10478 in ventricular CMs. (**A**). IV injection in *mdx* mice (n = 2 per aptamer) of 2 nmol (~50 μg) Cy3-labeled 2′F-Py RNA aptamer 10478, initial pool or mock injection (red) followed by collection of tissues 2 h after injection. Sections were immunostained with α-ACTN2 for CMs identification (green) and counterstained with Hoechst 33342 nuclear stain (blue). Representative images of left ventricles, where most CMs localize, were captured at the same laser intensities and acquisition time presented at (**A**). low magnification (×5 objective, scale bar = 200) and (**B**). High magnification (×20 objective, scale bar = 50 µm). Images were subjected to Deblurring on Zeiss Zen 3.4 (blue edition). (**C**). Quantitation of enrichment of the selected 2′F-Py RNA aptamer over the initial pool (T0) following IV injection of 5 nmol (~124 μg) and collection of selected organs after 2 h (n = 3 per aptamer). Quantities were calculated using the absolute quantification (RT-qPCR) standard curve method and expressed as fold change relative to the background binding detected with the initial pool (T0) used for selection (mean + SD; * *p* = non-significant). See also Statistical Analysis section in the Supplementary Materials. CMs, cardiomyocytes; gastro, gastrocnemius; quad, quadriceps. (**D**). Serum stability of the aptamer in 100% fresh mouse serum at 37 °C. Samples were first evaluated on a 12% (8 M Urea) denaturing PAGE gel at the indicated times (bottom). The serum stability curve (top) was generated by fitting the data in the equation of exponential one-phase decay on GraphPad Prism 8. Data were expressed as a percentage relative to the aptamer amount at time zero (0), n = 2.

As a consequence of its dominance in both NGS analyses, aptamer candidate 10478 was selected for individual assessments investigating cardiomyocyte enrichment following the in vivo SELEX adaptation described earlier. To this end, 10478 and the initial random pool were fluorescently labeled (red) and IV injected in *mdx* mice to assess their distribution to major organs. Figure 6A,B show a more selective localization of 10478 in left ventricular CMs (identified by ɑ-ACTN2 in green) than the initial pool (areas of colocalization are stained yellow). Additionally, the quantitative data obtained from RT-qPCR analysis (Figure 6C) of the entire CM pellet and other selected organs, further reinforce the result in Figure 6A,B with most signal being detected in the CM pellet and a small amount in other organs. A comparison of the relative enrichment of 10478 with that of pool 7 (Figure 3A) further shows that the aptamer candidate localizes to ventricular CMs 4.5 times more readily than the pool of sequences, suggesting selective CM localization for aptamer 10478.

As a last investigation, we assessed the rapid blood clearance of the aptamer following IV (indicated by the low 10,478 signal in kidneys, Figure 6B). Such rapid clearance could be explained by rapid renal excretion (especially when the aptamer size is below the cut-off limit of the kidneys), as well as nuclease degradation [10]. The nuclease stability assay in fresh mouse serum shows an almost 50% decrease in the first 24 h with minimal changes in the aptamer amount in the following 48 h (Figure 6D). While the 2′F-Py chemical modification is not as stable as the 2′-O-methyl modification for in vivo studies, in the context of our assessment it confers fairly good stability to the aptamer candidate 10478, against serum nucleases [24].

## 3. Discussion

The heart is probably the most complex and vital organ in the human body. It is composed of several different cell types that work in a highly organized manner to provide normal homeostasis. This unique yet overly complex morphology can obstruct many therapies directed specifically to only one cell type. The CMs, for example, that constitute most of the cell mass in reality account for only 30% of the total cell number in the heart [25]. Therefore, the therapeutic targeting of CMs for correction can be troublesome. One such case is DMD-related cardiomyopathy, with exon-skipping antisense oligonucleotide (AON) therapies currently demonstrating most promise [26]. Different strategies have been utilized to enhance the delivery of exon-skipping AONs to the heart muscle, such as adeno-associated viral vectors, liposomes, polymers, nanoparticles and cell-penetrating peptides [27,28,29,30,31,32]. However, none of these methodologies can specifically target the CMs for correction, leading to an increase in the administered dose, to compensate for the AON amount that is non-specifically adsorbed elsewhere [33]. Newer drugs would therefore be highly desirable to present with cell selectivity the ability to discriminate between, and so affect, only one cell population within a tissue for correction. In this work, we describe an adaptation of the in vivo SELEX approach for CMs targeting in an animal model of DMD. We demonstrate that an adaptation as simple as the incorporation of a cell-enrichment step, prior to the recovery of aptamers from tissues, can direct the selection towards a specific fraction of cells, the cardiac myocytes of the heart. Such aptamers could potentially be more suitable as targeting ligands for therapeutics requiring higher (targeting) precision, thus reducing the administered dose and dose-dependent toxicities. This is the first study to introduce a cell-enrichment step in a whole-animal selection platform. It is a merge between the previous golden standard aptamer methods, cell SELEX and in vivo SELEX, thus obtaining the advantages of both selection methods and potentially fewer of their disadvantages. The selective enrichment is achieved by digesting the tissue into a single cell population and subsequent cell isolation using a positive selection technique published in 2016 by Ackers-Johnson et al. [22]. Nevertheless, the cell-enrichment step can be applied to any organ, given that an appropriate enrichment strategy is available. For example, magnetic-bead cell separation or fluorescence-activated cell sorting could be used. Furthermore, magnetic-bead-based cell separation kits are nowadays commercially available for most cell types, providing simplicity and reproducibility to the cell-enrichment step [34]. The large size of CMs (60 µm) was a limitation in applying similar strategies to the selection of CM-targeted aptamers and at the same time the decisive factor for the isolation approach of choice. Another limiting factor was the fragility of isolated adult CMs, as changes in the environment (pH) and spontaneous contractility are likely to induce injury and cell death. A direct comparison between wild-type CM isolations and *mdx* CMs suggested some differences in the recovery yields. This is most likely correlated to the disease (DMD), where the decrease in dystrophin protein (a structural and protective muscle protein) renders *mdx* CMs more fragile, vulnerable to stress, and easily damaged [35].

Unlike previous in vivo SELEX studies that required 10–22 cycles to reach the enrichment plateau, our selection required as few as 7 [3,17,18,19,20]. This is most likely because the combination of intravenous administration with the cell-enrichment step makes the selection more stringent and directs the aptamers towards a specific target with simultaneous exclusion of sequences accumulating elsewhere, early on in the selection. Whether this type of selective enrichment has introduced bias into the overall approach is still unknown and should be further investigated.

While bioinformatics tools provide powerful analyses for the identification of potential therapeutic targets, conclusions are being drawn from large datasets of biological samples, computation models, algorithms and many more [1,36,37,38]. This could sometimes lead to results that might not fully agree with results obtained from wet-lab experiments, such as in the case of clusters UC18725 and UC11601 that showed no binding on heart sections following incubation. Another explanation could be the technique of choice. Incubation of aptamers on sections has been previously reported to sometimes yield variable results compared to when administered in vivo [39]. Furthermore, non-specific binding increases with incubation, thus potentially explaining the higher binding seen in liver and kidney sections when compared to their respective in vivo distribution results. While the latter has been a method commonly employed for several therapeutic molecules, perhaps the more recent in vivo imaging would have been an even better strategy to understand the true biodistribution and accumulation of the candidate aptamer in vivo rather than single-point fluorescence microscopy (that is prone to background fluorescence) [19,40,41]. This is a more dynamic approach that permits the examination of events related to normal physiology or disease in real time and is not limited to specific time points such as the 2 h used in this study. For instance, we could study the retention time of the aptamer in CMs and clearance rate without the need to sacrifice additional animals [42]. The low accumulation observed in the representative muscles, while not expected, could denote a common receptor with CMs, or that it is random accumulation due to the leaky nature of the muscle fibers in DMD [43]. In kidneys, liver and lungs the observed accumulation was expected, as they are highly perfused organs, with kidneys also being the preferential route of aptamer clearance [42]. While a more detailed investigation of the biodistribution is required, addition of polyethyl glycol molecules could be employed in future assessments to circumvent renal clearance [19,44]. On another note, using the peptide nucleic acid (PNA) hybridization assay or flow cytometry to quantitate the aptamers in tissues and CMs, respectively, rather than RT-qPCR (that is relatively prone to noise), could have been more illuminating, providing us with information such as the percentage of the injected dose that accumulated in each organ. For example, in the work by Biscans et al. (2021), 0.3% of the IV-injected siRNA dose was sufficient to induce silencing of *myostatin* and phenotypic changes in the heart for longer than one month [45]. Therefore, although there is CM enrichment, whether the actual amount that is retained is sufficient for therapeutic applications is as yet unknown.

## 4. Materials and Methods

### 4.1. Animals

Wild-type C57BL/10 mice and genetically dystrophic DMD*^mdx^* mice were bred in-house at the Transgenic Mouse Facility of the Cyprus Institute of Neurology and Genetics (registered breeder: CY/EXP/101). Six- to eight-week-old male mice were used. All animals were maintained under a 12-h light and dark cycle, provided with water and mouse chow.

### 4.2. Oligonucleotides

A 94-nucleotide single-stranded DNA (ssDNA) library containing a 40-base randomized region was synthesized as shown on Table S1 Fluorescein (FAM, green) or Cyanine 3 (Cy3, red) labeled aptamer pools (i.e., the initial pool and the enriched pool #7) were generated using the Silencer siRNA Labeling Kit with FAM or Cy3 dye (Thermo Fisher Scientific, Waltham, MA USA), respectively, as per the manufacturer’s instructions. Individual aptamer sequences were fluorescently labeled using the ULYSIS Nucleic Acid Labeling kit with Alexa Fluor 647 (U21660) or Alexa Fluor 594 (U21654) dye (Thermo Fisher Scientific, Waltham, MA, USA) as per the manufacturer’s instructions. All oligonucleotides (Table S1), including the ssDNA template, the individual aptamers, and the primer pairs were synthesized by IDT (Coralville, IA, USA) and delivered reconstituted in IDTE (pH 8.0) at 100 μM, as ssDNA. Unless in use, all oligonucleotides were aliquoted and stored at −20 °C.

### 4.3. Aptamer Library Preparation

The ssDNA template was converted to a double-stranded product using an annealing and elongation reaction as previously described [40]. Briefly, 1 nmol DNA template was incubated in the presence of 2 nmol SELEX Reverse primer (Table S1), 1.8 mM Tris-HCl pH8.0 and 1.5 mM MgCl_2_, and incubated at 95 °C for 5 min followed by 20 min at 20 °C. The PCR buffer at a final concentration of 1× was mixed with 200 µM dNTPs, Taq DNA polymerase (Qiagen, Hilden, Germany), in a separate tube and incubated at 95 °C for 5 min followed by 20 min at 23 °C. For the elongation of the library, the two reactions were mixed in one tube and incubated in the cycler for 30 min at 72 °C and 10 min at 25 °C. The corresponding 94 bp product was gel purified on a 10% native PAGE gel and used as template for in vitro transcription. The corresponding nuclease-resistant 2’F-Py RNA transcripts were produced following an overnight incubation at 37 °C, using the Durascribe T7 transcription kit (Lucigen, Middleton, WI, USA) as per the manufacturer’s instructions. To ensure transcription success, the control template included in the Durascribe T7 transcription kit was used at each transcription round. The control template is a 4.2-kb linearized DNA plasmid that produces a 1.4-kb 2’F-Py RNA transcript upon successful transcription. The size of the 2’F-Py RNA transcripts (77 b) was confirmed on a 6% denaturing (7M Urea) PAGE gel followed by gel extraction of the band. The product was then further purified using phenol–chloroform extraction and concentrated by ethanol precipitation. NanoDrop One Spectrophotometer (Thermo Fisher Scientific, Waltham, MA, USA) at A260 was used for quantification.

### 4.4. In Vivo SELEX

For each mouse, the 2’F-Py RNA aptamer library (Figure S1C) was folded in 115 µL DPBS buffer (Gibco, Dulbecco’s phosphate-buffered saline, no magnesium, no calcium, Thermo Fisher Scientific, Waltham, MA, USA) supplemented with 1 mM MgCl_2_, prior to the selection. The libraries were denatured at 70 °C for 10 min, snap cooled for 5 min at 4 °C and slowly refolded at 37 °C for 30 min, in a thermal cycler. The folded aptamers were systemically injected via the tail vein into three 8-week-old DMD*^mdx^* male mice (per round). After 2 h of circulation, the mouse chest was exposed and the CMs isolated from the heart as previously described. For each selection round, the CMs isolated from the injected mice (n = 3) were pooled and treated as one sample representative of the round. Total RNA extraction from primary CMs was extracted using TRIzol Reagent (Thermo Fisher Scientific, Waltham, MA, USA) as per the manufacturer’s instructions. To degrade the cellular RNA and recover only the 2’F-Py RNA aptamer libraries, total RNA was treated with RNase A (NEB, Ipswich, MA, USA) for 30 min at 37 °C, followed by phenol–chloroform extraction and ethanol precipitation. RNA samples from the selection rounds were stored at −80 °C.

To generate the pool for the next round, 0.5–1 μg total RNA was reverse transcribed into cDNA using the QuantiNova Reverse Transcription kit (Qiagen, Hilden, Germany). Half of the cDNA was amplified with the Q5 High-Fidelity DNA polymerase (NEB, Ipswich, MA, USA) in the presence of 3 µM selection primer mix (Table S1), 200 µM dNTPs, for 5 amplification cycles for the initial library and 22–30 cycles (Figure S1C) for the libraries recovered from the SELEX rounds. The cycling conditions were as follows: initial denaturation at 98 °C for 30 s; 5–30 cycles of denaturation at 98 °C for 30 s, annealing at 78 °C for 30 s, and extension at 72 °C for 30 s; and final extension at 72 °C for 2 min. The DNA template was gel purified and used as template to produce the 2’F-Py RNA transcripts for the next round of selection, as previously described. cDNA products and RNA transcripts from selection rounds were stored at −20 °C when not in use, in aliquots.

### 4.5. PCR Cycle Optimization

To prevent over-amplification of the pool, the number of PCR cycles was optimized at each round (Figures S1C and S6B). Briefly, 20 ng cDNA was amplified with the Q5 High-Fidelity DNA polymerase (NEB, Ipswich, MA, USA) in the presence of 0.4 µM selection primers mix (Table S1) and 200 µM dNTPs, for 5–30 amplification cycles with 2–5 cycle increments. The cycling conditions were the same as above. The products were then analyzed for aptamer recovery on a 10% native PAGE gel. PCR products from the selection rounds were kept at 4 °C for short-term storage or −20 °C for long term.

### 4.6. Next Generation Sequencing for Aptamer Enrichment

A modified 16S Metagenomic Sequencing Library preparation protocol for Illumina platforms was used to prepare the aptamer libraries for deep sequencing. Libraries were prepared by performing two subsequent overlapping PCR amplifications: Amplicon PCR and Index PCR (Figure S4). For the amplicon PCR, a 12.5 ng aliquot from each round was amplified using the KAPA HiFi HotStart Ready Mix PCR Kit (KR0370, Roche Basel, Switzerland), the NGS Forward and Reverse primers that are partially complementary to the aptamer fixed arms (Table S1), for ×8–10 cycles (cycle number was optimized for each sample, to reduce over-amplification). The annealing temperature and time were optimized to 74 °C for 25 s, respectively. For the Index PCR, 50 ng gel-extracted Amplicon PCR product was amplified for additional 10 cycles, using the HiFi HotStart Ready Mix PCR Kit (KR0370, Roche, Basel, Switzerland) and the Nextera XT Index primer 1 (N7XX) and Index primer 2 (S5XX) (Nextera XT Index kit, 24 indexes, 96 samples, FC-131-1001, Illumina, San Diego, CA, USA) that were partly complementary to the sequence of the NGS primers (Table S1). For each round a different index combination was used to assist the deconvolution process of the data following the NGS run (Table S2). For the index PCR, the manufacturer’s suggested cycling conditions were used.

The concentrations of the final products were determined using Qubit 2.0 Fluorometer and the Qubit dsDNA HS Assay kit (Q32851, Thermo Fisher Scientific, Waltham, MA, USA). Aliquots from each round were pooled together and further diluted as per the manufacturer’s instructions (16S Metagenomic Sequencing Library Preparation, Illumina) for sample loading on the sequencing platform. Sequencing was done on Illumina MiniSeq platform using the Miniseq Mid Output Kit (300-cycles) (FC-420-1004, Illumina, San Diego, CA, USA) in duplex read mode for 2 × 150-cycles with the addition of 9% PhiX in the run as loading control. Aliquots from each round before the pooling were kept as backup at −20 °C.

### 4.7. Bioinformatics Analysis of NGS Data

Raw sequencing data were demultiplexed (assigning the sequence reads to separate files for each index tag/sample) and fastq data files were generated using the MiniSeq onboard data analysis software, bcl2fastq2 conversion software v2.20 (see also Figure 4A). Next, demultiplexed reads from each SELEX round were trimmed to remove any Illumina library adapters and indices remaining after the MiniSeq filtering step using Trimmomatic v0.39 with default settings and the included NexteraPE-PE.fa file as a reference adapter library (passed to the ILLUMINACLIP attribute) [46,47,48]. Next, using the same approach, the constant regions were trimmed by including the corresponding sequences in the reference library file. Simultaneously an average quality threshold of 28 was applied to the resulting reads. The lengths of the variable region of the reads ranged from 39–41 bp and were clustered using CD-HIT v4.8.1 program where a 90% sequence-similarity threshold was set. For each cluster, the total and representative sequence read counts were recorded.

Cluster analysis was performed using custom written scripts in R V3.6.1 language. Unique/Enriched cluster ratios and base pair compositions were analyzed using the biostrings, stringr, shortread, igraph packages (https://igraph.org/, accessed on 13 May 2020) and visualized using the ggplot2 package [49]. We classified as unique and enriched the clusters comprising fewer or more than 50 sequences, respectively. The unique and enriched fractions (Figure 4B) were calculated as the sum of the read counts in each class over the total number of reads in the dataset, per round. The fraction of read counts (Figure 4D) was derived by binning the sequences with respect to the read counts of each across 6 bands (≤10, 11–100, 101–1000, 1001–5000, 5001–10,000, >10,000) and visualized using ggplot2 package in R. Nucleotide ratios per position (Figure 4C) were obtained by calculating the frequency of each base per position over the total number of reads. Diversity indices (Figure S5) were calculated as follows (where R is number of different sequences observed and $p_{i}$ their relative abundance):

$$Richness,R=\sum_{i=1}^{R}p_{i}^{0}$$

$$Shannon\;Index,H=-\sum_{i=1}^{R}p_{i}\;ln\;ln\;p_{i}$$

$$Inverse\;Simpson,S^{-1}=\frac{1}{\sum_{i=1}^{R}p_{i}^{2}}$$

Tracking of the clusters across cycles was performed by assigning a unique cluster ID at the round of its first appearance. The representative sequence from each unique cluster at a given round was mapped to the next round by sequence alignment using the Needleman–Wunsch algorithm in the biostrings package in R and applying a score threshold of 30. Representative aptamer sequences not aligned sufficiently with any sequence of the next round (alignment score < 30) were assumed to be lost. Tracked clusters were visualized using ggplot2 and igraph (Figure 4E and Figure 5). Selected aptamer sequences shown in Figure S7 were further analyzed using the multiple sequencing alignment tool Clustal Omega (https://www.ebi.ac.uk/Tools/msa/clustalo, accessed on 5 June 2020) and the in-built phylogenetic tree tool.

### 4.8. RT-qPCR Quantification

For the relative gene expression method of analysis used to assess aptamer pool binding/internalization, total RNA was extracted from CMs from the selection rounds, using TRIzol Reagent (Thermo Fisher Scientific, Waltham, MA, USA), and converted to cDNA using the QuantiNova Reverse Transcription kit (Qiagen, Hilden, Germany) as per the manufacturer’s instructions. Then, an RT-qPCR reaction was carried out with SELEX primers and 18S rRNA primers (Table S1), using the QuantiNova SYBR Green master mix (Qiagen, Hilden, Germany). All reactions were performed on the Qiagen Rotor-Gene Q Series using the 72-well high-throughput rotor, 0.1 mL strip tubes (Qiagen, Hilden, Germany) and a final reaction volume of 20 µL. A master mix was prepared for each primer pair target comprising 1× QuantiNova SYBR Green master mix, 0.7 µM forward and reverse primer mix, and nuclease-free water. Input cDNA template was 10 ng for SELEX primer and 0.04 ng (0.0367 ng) for 18S rRNA, in a final volume of 4 µL. For the no template control (NTC), 4 µL nuclease-free water was used as template. The run was performed as a 3-step protocol with an initial hold at 95 °C for 2 min (initial denaturation), followed by 40 cycles of a denaturation step at 95 °C for 5 s and a combined annealing/extension step at 65 °C for 25 s with data acquisition at Green channel. To assess the primer specificity, a dissociation melt curve was performed at the end of the run between 72–95 °C with a 1 °C increment and 5 s hold between steps. Gain optimization before melt was selected. All reactions were performed in technical duplicates and biological triplicates (n = 3). Data analysis was performed using the relative gene expression method (ΔΔC_T_) [50]. Using the ΔΔC_T_ method, data are presented as the fold change in gene expression (i.e., SELEX primers) normalized to the endogenous reference gene (i.e., 18S rRNA primers) and relative to the calibrator (i.e., Round 1). For Round 1, ΔΔC_T_ equals zero and 2^0^ equals one, so that the fold change in gene expression relative to Round 1 equals 1, by definition. For all other samples (i.e., Rounds 2, 4–7), evaluation of ΔΔC_T_ indicates the fold change in binding/internalization relative to Round 1.

The enrichment in the initial pool (T0), enriched pool #7 (T7) or 2′F-Py RNA aptamers 10478 following IV injections of 5 nmol (~124 μg) was determined using the absolute quantification (standard curve) method. In short, a standard curve was constructed using a 10-fold serial dilution of known aptamer concentrations varying from 10^2^ to 10^−3^ ng/reaction as shown in Figure S2. The quantification cycle (Ct) values were then plotted against the log[quantity] of the serial dilutions. Aptamer quantity in unknown samples was interpolated by fitting their Ct values on the standard curve. For the distribution assays, relative enrichment was determined as follows: the aptamer quantity was first normalized to the tissue mass (i.e., ng aptamer/mg of tissue) and then expressed relative to the background binding detected with the initial pool (T0) of sequences used for the selection. The tissue mass of each organ was experimentally determined (Table S4).

$$Relative\;Enrichment=\frac{ng/mg\;of\;tissue\;of\;T7\;enriched\;pool\;or\;aptamer}{ng/mg\;of\;tissue\;of\;inital\;T0\;pool}$$

$$Relative\;binding=\frac{ng\;of\;aptamer\;detected\;in\;RT-qPCR}{ng\;of\;input\;cDNA\;in\;RT-qPCR}$$

All reactions were performed as previously described using the Quantinova RT kit for cDNA synthesis (as per the manufacturer’s instruction) and the QuantiNova SYBR Green master mix (Qiagen, Hilden, Germany). Runs were performed on the Qiagen Rotor-Gene Q as previously, at a final reaction volume of 15 µL. A master mix was prepared using the qPCR SELEX primers (Table S1) comprising 1× QuantiNova SYBR Green master mix, 0.3 µM forward and reverse primer mix, and nuclease-free water. Input cDNA template was (experimentally determined to) 50 ng in a final volume of 5 µL. For the no template control (NTC), 5 µL nuclease-free water was used as template. The cycling conditions of the run and melt-curve analysis were as described above. All reactions were performed in technical duplicates and biological triplicates (n = 3). All cDNA samples were stored at 4 °C for short-term storage and −20 °C for long term.

### 4.9. Immunocytochemistry

To assess the purity of the isolated CM populations at the initial steps of the study, cells were cultured on laminin treated plates and immunostained with the CM-specific marker, alpha sarcomeric actinin 2 (α-ACTN2, A7811, Sigma-Aldrich, St. Louis, MO, USA) at a 1:1000 dilution, as previously described. Goat anti-rabbit Alexa Fluor 488 Plus secondary antibody (A32731, Invitrogen, Carlsbad, California) was used at a final concentration of 10 µg/mL. Cells were counterstained with Hoechst 33342 nuclear dye at a dilution of 1:2000 dilution in PBS for 15 min (H370, Invitrogen, Carlsbad, CA, USA).

### 4.10. Tissue Harvesting and Preparation

Two hours after the injection, DMD*^mdx^* mice were intracardially (left ventricle) perfused with 20 mL cold DPBS and then 20 mL 4% paraformaldehyde (powder) in PB buffer. Next, the heart was harvested and further fixed at 4 °C (≥12 h) followed by cryoprotection in 30% sucrose in PBS for an additional 12–18 h (overnight) at 4 °C. The samples were removed from the sucrose solution, washed thrice in PBS for 5 min and then, tissue blocks were prepared by embedding the tissue in Tissue Tek O.C.T. (V.W.R. Chemicals, Radnor, PA, USA) in single-use cryomolds that were partially dipped in liquid-nitrogen-cooled isopentane. Samples were immediately stored in a −80 °C freezer, in appropriate airtight containers. Using a cryostat, 10–12 µm thick sections were produced and mounted on SuperFrost Plus adhesive slides (Thermo Fisher Scientific, Waltham, MA, USA). To visualize the CMs, longitudinal heart sections were generated. Before storing the slides in a −80 °C freezer, they were allowed to dry at room temperature for 20–30 min, protected from light.

For C57BL/10 (WT) mouse hearts (Figure S3) as well as for the major organs (heart, liver and kidneys) collected for the ex vivo experiments in Figure S6, mice were perfused with 20 mL cold PBS and then the organs were harvested and snap-frozen in liquid-nitrogen-cooled isopentane and stored at −80 °C in a freezer in airtight containers. Tissue sections were prepared on a cryostat as described in the previous section.

### 4.11. Tissue Processing and (Immuno)Staining

To identify the CMs on heart sections from DMD*^mdx^* or C57BL/10 mice that were previously IV injected for 2 h with fluorescently labeled aptamer pools (T0 or T7), the selected 2′F-Py RNA aptamer 10478 or mock injection heart sections were incubated with 0.1% Triton X-100 in PBS for 15 min. Samples were then blocked for 45 min in blocking buffer (5% BSA, 0.1% PBS-Triton X-100 in PBS) followed by overnight incubation with the primary antibody (mouse α-ACTN2, A7811, Sigma-Aldrich, St. Louis, MO, USA) diluted in 1:200 or 1:400 in blocking buffer in a humidity chamber at 4 °C. The next day, three 5-min PBS washes were followed by a 1-h incubation with the secondary antibody, goat anti-mouse Alexa Fluor Plus 488 or goat anti-mouse Alexa Fluor 594 (Invitrogen, Carlsbad, CA, USA) diluted 1:100 or 1:200 in blocking buffer at room temperature.

For the ex vivo assessment of additional selected aptamer candidates (Figure S6), heart, liver and kidney sections from DMD*^mdx^* mice were fixed with 4% PFA in PB buffer for 10 min, followed by 3× PBS washes and incubation with 200 nM Alexa Fluor 647-labeled aptamers at 37 °C for 1 h.

All sections were counterstained using 1 µg/mL Hoechst 33342 (Invitrogen, Carlsbad, CA, USA) in PBS for 10 min, to identify the nuclei, washed twice with PBS, air-dried briefly and mounted using Dako fluorescence mounting medium (Agilent, Santa Clara, CA, USA). The edges were sealed and stored at 4 °C, protected from light.

### 4.12. Fluorescence Microscopy

Fluorescent microscopy images were obtained at the same laser intensities and exposure times using either a Nikon Eclipse Ni microscope (×20 objective) or a Zeiss Axio Observer Z1 inverted microscope (×10 and ×20 objective). Images obtained with the latter were further processed on the Zeiss Zen 3.4 (blue edition) software to improve their clarity via the “Deblurring” function.

### 4.13. Serum Stability Assay

To assess serum stability, 50 pmol 2′F-Py RNA aptamer 10478 was incubated in 100% freshly collected mouse serum from C57BL/10 mice. Briefly, whole blood was collected by terminal cardiac puncture, allowed to clot at room temperature for 20 min and then the serum was separated by centrifugation at 2000× *g* for 20 min at 4 °C. Fifty picomole aptamer in 10 microliters = 5 pmol/µL) was added to 90 µL serum and incubated at 37 °C for up to 72 h. At each time point, the aptamer was recovered by methanol–chloroform extraction as previously described [51]. Samples were stored at −80 °C until all time points were collected. Then an aliquot of 10 µL was diluted 1:1 in Gel loading buffer II (Thermo Fisher, Waltham, MA, USA), denatured at 95 °C for 5 min and run on a 12% (8 M Urea) denaturing PAGE gel for 25 min and stained with SYBR Gold Nucleic Acid Gel Stain (Thermo Fisher Scientific, Waltham, MA, USA) at 1:10,000 in 1× TBE buffer for 15 min with constant shake. Bands were visualized by UV exposure and then their intensities (OD values) quantified with ImageJ software v.1.8.0 and expressed as percentage (%) relative to the amount at time zero (0). The graph was plotted on GraphPad Prism v8 by fitting the data in a one-phase exponential decay model.

### 4.14. Secondary Structure Prediction

All RNA sequences are reported in Supplementary Materials (Table S1). Aptamer secondary structures were predicted using the NUPACK web application (http://www.nupack.org/, accessed on 28 May 2020) at default settings.

### 4.15. Statistical Analysis

For each analysis, three independent experiments were conducted unless otherwise specified. Mean, SD and graphs were determined/plotted using Microsoft Excel 2013 and/or GraphPad Prism 7, unless otherwise stated. To study the significance of the observed fold change (FC) (Figure 3A and Figure 6C), a statistical analysis was performed using a one-sample *t* test (https://www.graphpad.com/quickcalcs/oneSampleT1/, accessed on 16 August 2023) on log-transformed data [log2(FC)] [52]. Significance was set at a *p* value of ≤0.05.

### 4.16. Terms and Definitions

A glossary of terms and definitions is included in Appendix A.

## 5. Conclusions

In conclusion, this study presents a potentially new selection approach where 2′F-Py RNA aptamers could discriminate between the different cells of the heart. It is an adaptation of the in vivo SELEX, and the design concept is demonstrated by directing the selection of aptamers towards the ventricular CMs in the heart of *mdx* mice. As this work shows the design of a cell-specific approach in the heart for the first time, there is more to be done in terms of refinement of the method. This would include characterization of internalization, endosomal escape, molecular target(s), and potential cross-species reactivity and receptor recognition (i.e., similarities and/or differences) between mouse and human CMs that could demonstrate utility as targeting ligands as well. If successful, our approach holds promise for the therapy of muscular dystrophies among other diseases. Finally, the concept of cell enrichment could be widely applied to any in vivo SELEX design where the target requires cell-level specificity, be that for diagnostic or therapeutic purposes.

## Figures and Tables

**Table 1:** CMs recovery from C57BL/10ScSn-*Dmd^mdx^*/J mice.

Gender	Male
Age	8 weeks old
Average yield	5.53 × 10^5^ ± 3.79 × 10^4^
Average viability	81.7 ± 7.64

Data presented as mean ± SD, n = 3. Cells were counted using the Trypan Blue exclusion assay.

## Data Availability

Data is contained within the article.

## Supplementary Materials

The following supporting information can be downloaded at: https://www.mdpi.com/article/10.3390/ph16091264/s1, Figure S1: Library Preparation and SELEX monitoring; Figure S2: Identification of the linear dynamic range of RT-qPCR for absolute determination of aptamer amount; Figure S3: Aptamer pool localization in healthy, ventricular CMs; Figure S4: Aptamer libraries preparation for NGS analysis; Figure S5: Enrichment of CM-enriched aptamers via NGS; Figure S6: Assessment of additional aptamer candidates; Figure S7: Predicted structures; Figure S8: Confirmatory rounds. In vivo SELEX conditions and indirect monitoring of aptamer recovery via PCR; Figure S9: Confirmation of cardiomyocyte-targeted 2’F-Py RNA aptamer enrichment; Figure S10: Aptamer cluster tracking across SELEX and frequencies of specific candidates; Table S1: List of oligonucleotide sequences and primer pairs; Table S2: Nextera XT adapter combinations for NGS samples; Table S3: Frequency of selected clusters in Round 7; Table S4: Average body weight and organ weights in C57BL/10ScSn-Dmdmdx/J. Supplemental statistical analysis data.

## Appendix A

**Table A1:** Glossary of Terms and Definitions.

UCID	Unique cluster identification number assigned to the NGS data, abbreviated UC# that allows the identification of the different clusters/families of sequences in the NGS data following SELEX.
Clusters	Groups or families of biological sequences that share similarities in their primary sequence. Sequence-clustering algorithms attempt to group the sequences that are somewhat related and the most represented sequence serves as the seed sequence of the cluster.
Unique sequences	Any sequence in the NGS data that presents with ≤50 read counts.
Enriched sequences	Any sequence in the NGS data that presents with >50 read counts.
Number of raw sequences	The total number of reads going off the sequencer and into data analysis.
Read count	The number of reads/sequences going off the sequencer or that align to a reference sequence (e.g., to the seed sequence of a cluster).
Read count fraction	This is the proportion of the sum of the read counts (sequences) in each class (enriched or unique) over the total number of reads in the dataset, per round. Total read in the dataset = 1.
Bin fraction	Bin fraction denotes the proportion of each bin over the total population of reads on the dataset. Total reads = 1. The fraction of read counts was derived by binning the sequences with respect to the read counts of each across 6 bands (≤10, 11–100, 101–1000, 1001–5000, 5001–10,000, >10,000).
Base fraction	Nucleotide (base) fractions per position, in a given round, were obtained by calculating the frequency of each base per position over the total number of reads. Total number of reads = 1.
Reads per million (RPM)	The read counts per cluster were divided by the “per million” scaling factor. This normalizes for sequencing depth, giving the reads per million. The “per million” scaling factor is derived by counting the total reads in a sample (i.e., SELEX round) and then dividing that number by 1,000,000.
Technical duplicate	Technical replicates are repeated measurements of the same sample that demonstrate the variability of the protocol. Technical replicates are important because they address the reproducibility of the assay or technique. In this study, a technical duplicate is used in RT-qPCR assay to ensure the validity of the method (i.e., the pipetting technique). It is the same cDNA pipetted into multiple wells, thus Ct values with little variability should be obtained. The mean value from these replicates is then used as a representative value for each biological sample in subsequent data analyses.
Biological replicate	Biological replicates are parallel measurements of biologically distinct samples that capture random biological variation, which can be a subject of study or a source of noise itself. Biological replicates address how widely your experimental results can be generalized. Unless otherwise stated, three biologically distinct samples (n = 3) were used in each experiment (biological triplicate).

## Appendix B

**Table A2:** The top 10 aptamer clusters identified in each SELEX round.

SELEX Round	Ranking	UCID	Cluster ID	Cluster Counts	Top Seq Counts	SequenceID	Random Region Sequence
1	1	UC145	Cluster 144	396	345	>2-345-2257.31_2	AGGGTAAGCCTTTCCATCGGGTCGACTTCGGATTGCATCG
	2	UC156	Cluster 155	394	346	>1-346-2263.85_1	TGTGAGTGATTACGCTCTGTGCGTATGGGGACAGTTCCGC
	3	UC538	Cluster 537	386	333	>3-333-2178.79_3	AAAGTCTACAGGTGAAAGGCGTCACCGCGAGGCGAGCGTT
	4	UC124	Cluster 123	384	326	>5-326-2132.99_5	CGGTGCACTGGCATGCTGGACCGGAGGTCAGGACGGTCGG
	5	UC17	Cluster 16	381	323	>6-323-2113.36_6	TGGCCCGCTACTCCGCGGTCTATACTAGTATTCCGTAACA
	6	UC45	Cluster 44	378	323	>6-323-2113.36_7	ACTGTGTCGATCAGGTAAACGACACTTGCGGCCTGCTATA
	7	UC88	Cluster 87	374	321	>8-321-2100.28_8	TACCCCATAATAGGCCTTGTAGGATCGTAGACGTTACGTC
	8	UC504	Cluster 503	374	314	>13-314-2054.48_13	TACTTGACAACACTAGTGATAGCAGAATCGCGAGACCGCA
	9	UC400	Cluster 399	371	327	>4-327-2139.53_4	GTGGACGAGCCGGGCATGGTCGAGTGTGAAGGGAGCCGCG
	10	UC605	Cluster 604	370	321	>8-321-2100.28_9	CCGGCGACTCTCGCGAACAGCTTCCCATCCGCATTTGTGG
2	1	UC3215	Cluster 141	723	635	>1-635-4884.31_1	TGCCGCAGGGTGTGGATTGAATTGACGGTGAGACGCGCAC
	2	UC7082	Cluster 35	718	631	>2-631-4853.55_2	CGGTGGACGTGTAGCGGGAATCCGCGGCAAACACAGAGCT
	3	UC7117	Cluster 75	717	615	>5-615-4730.48_5	GGCCCAACGTGGTTGGGGTCAACACGCGGGATTCGGGGTT
	4	UC7087	Cluster 40	705	609	>7-609-4684.33_7	ATAGCGTCCGGCTAGGCTTTCTCGGTGCGCAGCGGAGACA
	5	UC7091	Cluster 45	701	617	>4-617-4745.86_4	GCACTTGCAGCGCGGTGTACGCTAACGCCTGGGCCGGTGA
	6	UC7171	Cluster 135	690	622	>3-622-4784.32_3	TCTGTGCACGGCATCCGCTTAGAGTGTCCGGTCGGACATC
	7	UC3458	Cluster 269	689	610	>6-610-4692.02_6	AGGGCGGGTCGCGGGCCTGGTGATTGGACGGAGGCTGGCC
	8	UC7116	Cluster 74	683	606	>8-606-4661.25_8	CGATGCCCTGTGGTCGGTCGCCCGGCAGGGCTGTGCAGTT
	9	UC7053	Cluster 2	677	578	>15-578-4445.88_15	CGGTTCCGAGCGTTGGTGGAGGACGCGGGTAGGCGGACGT
	10	UC7189	Cluster 155	677	585	>11-585-4499.72_11	TGAGCCTGCGCGCGGGGGGAGGCGGCGGAGGACCAGTAGT
3	N/A—No back up sample for NGS analysis						
4	1	UC10474	Cluster 2	9235	8057	>1-8057-43057.24_1	CGACGGACAAGGCTCACCGTGGCCATGTGAGCTCGGGCGC
	2	UC10476	Cluster 5	9086	8034	>2-8034-42934.33_2	TGCGACGTGGGCGCGTCATGCTGCGCGGTGCTGTGCACGC
	3	UC1370	Cluster 3	8256	7138	>3-7138-38146.03_3	ACGGGCGCCCGTGCATAAGGTGCGGCGGGCTGACGTGTCG
	4	UC10479	Cluster 8	7072	5973	>4-5973-31920.18_4	CACGCGGCGGCCGTGAATGGTCACGGAGGCGAGCTGTGCC
	5	UC10478	Cluster 7	5617	4911	>5-4911-26244.77_5	TGCAGGTGCATGTGGGATCACGCGCGGTTAGGTCGCCGCG
	6	UC10483	Cluster 12	5143	4463	>6-4463-23850.62_6	TCACGGGCGTGGCGGGCGACGAGCCACGGAGCGGGGTTGC
	7	UC10984	Cluster 547	4316	3932	>7-3932-21012.92_7	ATGTCACGAACGAGGGCGTGCTCGCGTGGTGCGGAGGCA
	8	UC10488	Cluster 17	4190	3664	>8-3664-19580.70_8	GTGCGCCACAGGTGTTACGGTGGTGCATCCGTGGGCTGCG
	9	UC10481	Cluster 10	3752	3309	>9-3309-17683.56_9	CGGAGCCACCGGCGCGTGGGTGCGGGTGCGGCCACCAGCA
	10	UC10487	Cluster 16	3528	3109	>10-3109-16614.74_10	TGACGGCCCTGCAAGGAGGGCTAGGATGTCGCTGTTGCGC
5	1	UC10478	Cluster 4	10293	8302	>1-8302-56908.23_1	TGCAGGTGCATGTGGGATCACGCGCGGTTAGGTCGCCGCG
	2	UC11601	Cluster 5	9563	8251	>2-8251-56558.64_2	CGACGGACAAGGCTCACCGTGGCCATGTGAGCTCGGGCGC
	3	UC10476	Cluster 9	7566	6655	>3-6655-45618.44_3	TGCGACGTGGGCGCGTCATGCTGCGCGGTGCTGTGCACGC
	4	UC1370	Cluster 7	7499	6515	>4-6515-44658.77_4	ACGGGCGCCCGTGCATAAGGTGCGGCGGGCTGACGTGTCG
	5	UC10481	Cluster 8	7128	6262	>5-6262-42924.52_5	CGGAGCCACCGGCGCGTGGGTGCGGGTGCGGCCACCAGCA
	6	UC10984	Cluster 428	5679	5206	>6-5206-35685.89_6	ATGTCACGAACGAGGGCGTGCTCGCGTGGTGCGGAGGCA
	7	UC10479	Cluster 10	3747	3314	>7-3314-22716.68_7	CACGCGGCGGCCGTGAATGGTCACGGAGGCGAGCTGTGCC
	8	UC16931	Cluster 12	3223	2828	>8-2828-19385.27_8	TGGGGGCTCAGTGACGGCGCGTCGTCGTTGAGCAGCGGCA
	9	UC10484	Cluster 11	3070	2545	>9-2545-17445.37_9	CGCGGCCCCGGTAGTGTGGCTGGAGGGGTTGTTGTCGACA
	10	UC10494	Cluster 2	3057	2508	>10-2508-17191.74_10	CGTGGGACGGCCGGCGTAGGGTCGGCAGCGAGTGGCGCGC
6	1	UC10478	Cluster 3	10172	8763	>1-8763-61923.64_1	TGCAGGTGCATGTGGGATCACGCGCGGTTAGGTCGCCGCG
	2	UC11601	Cluster 4	7883	6702	>2-6702-47359.61_2	CGACGGACAAGGCTCACCGTGGCCATGTGAGCTCGGGCGC
	3	UC1370	Cluster 1	7636	6472	>3-6472-45734.31_3	ACGGGCGCCCGTGCATAAGGTGCGGCGGGCTGACGTGTCG
	4	UC10984	Cluster 311	6694	6016	>4-6016-42512.00_4	ATGTCACGAACGAGGGCGTGCTCGCGTGGTGCGGAGGCA
	5	UC10476	Cluster 7	6010	5149	>5-5149-36385.35_5	TGCGACGTGGGCGCGTCATGCTGCGCGGTGCTGTGCACGC
	6	UC18725	Cluster 10	5056	4306	>6-4306-30428.30_6	CGGAGCCACCGGCGCGTGGGTGCGGGTGCGGCCACCAGCA
	7	UC17023	Cluster 8	4858	4173	>8-4173-29488.46_8	CACGCGGCGGCCGTGAATGGTCACGGAGGCGAGCTGTGCC
	8	UC18722	Cluster 5	4856	4182	>7-4182-29552.06_7	TCACGGTGGGATGACTGAAGGTCTGGTGCGACCGGGGCGC
	9	UC10489	Cluster 17	3427	2949	>9-2949-20839.07_9	GGCGCGCCAGTCGCTCCGAGGGAGGGTGCGACGGTGCGTC
	10	UC16938	Cluster 15	2911	1953	>12-1953-13800.85_12	CACGGCAACTGTGAGGCAAAAACGCCTTTGGCCCGGCGCT
7	1	UC10478	Cluster 10	10716	9067	>1-9067-52008.17_1	TGCAGGTGCATGTGGGATCACGCGCGGTTAGGTCGCCGCG
	2	UC10476	Cluster 3	8321	6970	>2-6970-39979.81_2	TGCGACGTGGGCGCGTCATGCTGCGCGGTGCTGTGCACGC
	3	UC18725	Cluster 8	7826	6729	>4-6729-38597.44_4	CGGAGCCACCGGCGCGTGGGTGCGGGTGCGGCCACCAGCA
	4	UC10984	Cluster 263	7715	6915	>3-6915-39664.33_3	ATGTCACGAACGAGGGCGTGCTCGCGTGGTGCGGAGGCA
	5	UC11601	Cluster 2	7300	6184	>5-6184-35471.33_5	CGACGGACAAGGCTCACCGTGGCCATGTGAGCTCGGGCGC
	6	UC1370	Cluster 11	6511	5386	>7-5386-30894.01_7	ACGGGCGCCCGTGCATAAGGTGCGGCGGGCTGACGTGTCG
	7	UC18776	Cluster 11	6511	5386	>7-5386-30894.01_7	ACGGGCGCCCGTGCATAAGGTGCGGCGGGCTGACGTGTCG
	8	UC16938	Cluster 35	3812	2578	>12-2578-14787.37_12	CACGGCAACTGTGAGGCAAAAACGCCTTTGGCCCGGCGCT
	9	UC20614	Cluster 5	3516	3017	>10-3017-17305.46_10	CTGCCGGCGGTTGGGCCCTGGGCGGGCCAGCGGATGTCGC
	10	UC16931	Cluster 14	3437	2860	>11-2860-16404.91_11	TGGGGGCTCAGTGACGGCGCGTCGTCGTTGAGCAGCGGCA

Notes: 1. UCID: Unique cluster identification number. 2. Cluster counts: How many reads (counts) are in a cluster (i.e., cluster size) prior to RPM normalization. 3. Top Seq. Count: How many times the most represented sequence was read/counted in the cluster before RPM normalization. 4. Sequence: The seed sequence of the cluster. 5. Enriched clusters are highlighted with the same background color through the SELEX rounds, thus making their ranking among the top 10 sequences, easily recognized.
