# Supplementary material for: Selective Delivery to Cardiac Muscle Cells Using Cell-Specific Aptamers
Source: Pharmaceuticals (Basel). 2023 Sep 6;16(9):1264. doi: 10.3390/ph16091264 (PMC10534653; doi:10.3390/ph16091264)
Supplement: Supplementary file 1 [file pharmaceuticals-16-01264-s001.zip › Pharmaceutical Supplementary Information.pdf]

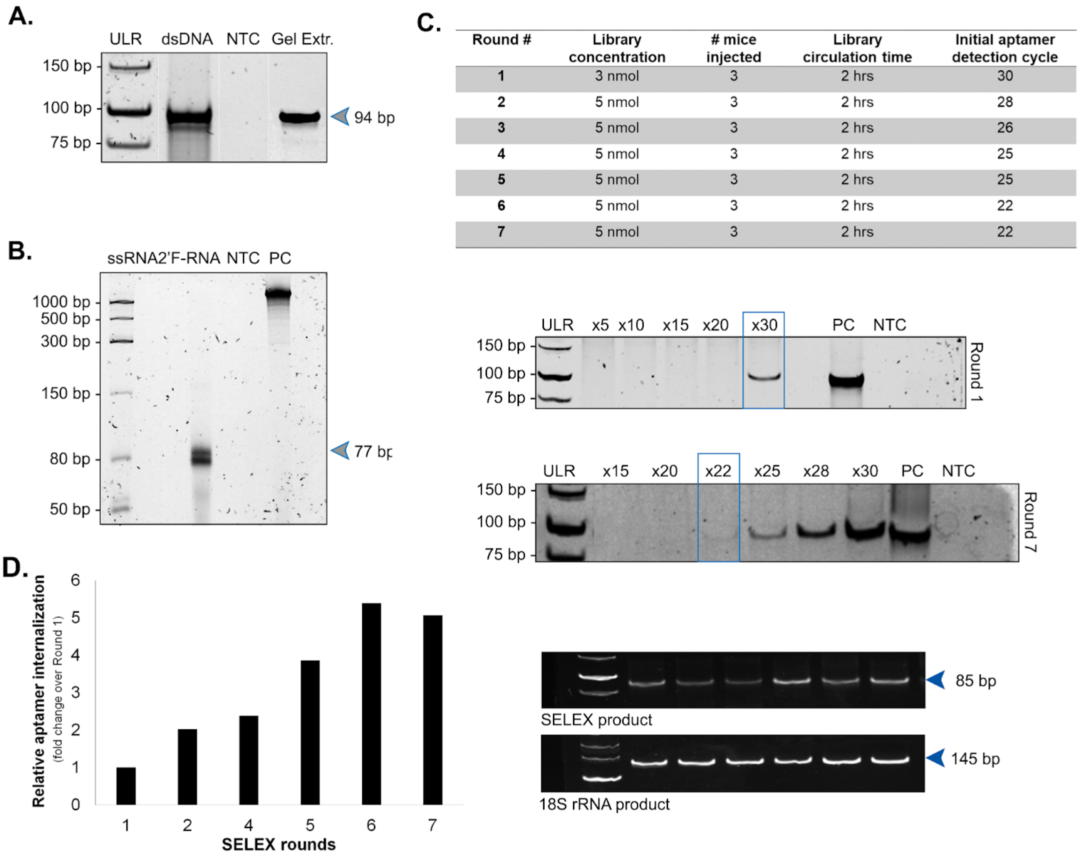

Round #	Library concentration	# mice injected	Library circulation time	Initial aptamer detection cycle
1	3 nmol	3	2 hrs	30
2	5 nmol	3	2 hrs	28
3	5 nmol	3	2 hrs	26
4	5 nmol	3	2 hrs	25
5	5 nmol	3	2 hrs	25
6	5 nmol	3	2 hrs	22
7	5 nmol	3	2 hrs	22

Figure S1. Library Preparation and SELEX monitoring, Related to Figure 1. A. Native polyacrylamide gel showing successful conversion of the initial ssDNA template to double stranded product (94 bp), before (lane 2) and after gel extraction (lane 4). **B.** Denaturing polyacrylamide gel showing successful conversion of the dsDNA product in **A.** to the corresponding initial 2'F-Py RNA aptamer pool (lane 3, 77 b). Modified bases (2'fluoropyrimidines, 2'F-Py) were incorporated in the transcription reaction to confer nuclease resistance to the library. PC corresponds to the Durascribe T7 Transcription kit positive control (PC) DNA template that produces a 1.4-kb corresponding 2'F-Py RNA transcript (see also Materials and Methods). **C.** *In vivo* Cell SELEX conditions and indirect monitoring of aptamer recovery via PCR. **Bottom.** Representative polyacrylamide gels of indirect monitoring of aptamer recovery (94 bp PCR product) from CMs for selection rounds 1 and 7. Recovery was assessed via conventional PCR utilizing primers specific for the initial pool. NTC: No template control; PC: Positive control; ULR: Ultra low range DNA ladder; ssRNA: Low range ssRNA ladder; x5 - x30: denote PCR cycles. **D.** Quantitation of aptamer pool binding/internalization in CMs via RT-qPCR, using the relative gene expression method of analysis. Data were normalized to 18S rRNA and expressed as fold change relative to Round 1 enrichment. For each round, recovered CMs from three mice were pooled together to create a pool representative of each round and then an aliquot from this representative pool was used for the RT-qPCR assessment. **Right.** Native polyacrylamide gel confirmation of RT-qPCR products (85 bp for SELEX RT-qPCR primers and 145 bp for 18S rRNA). All gel images were subjected to cropping for purposes of clarity.

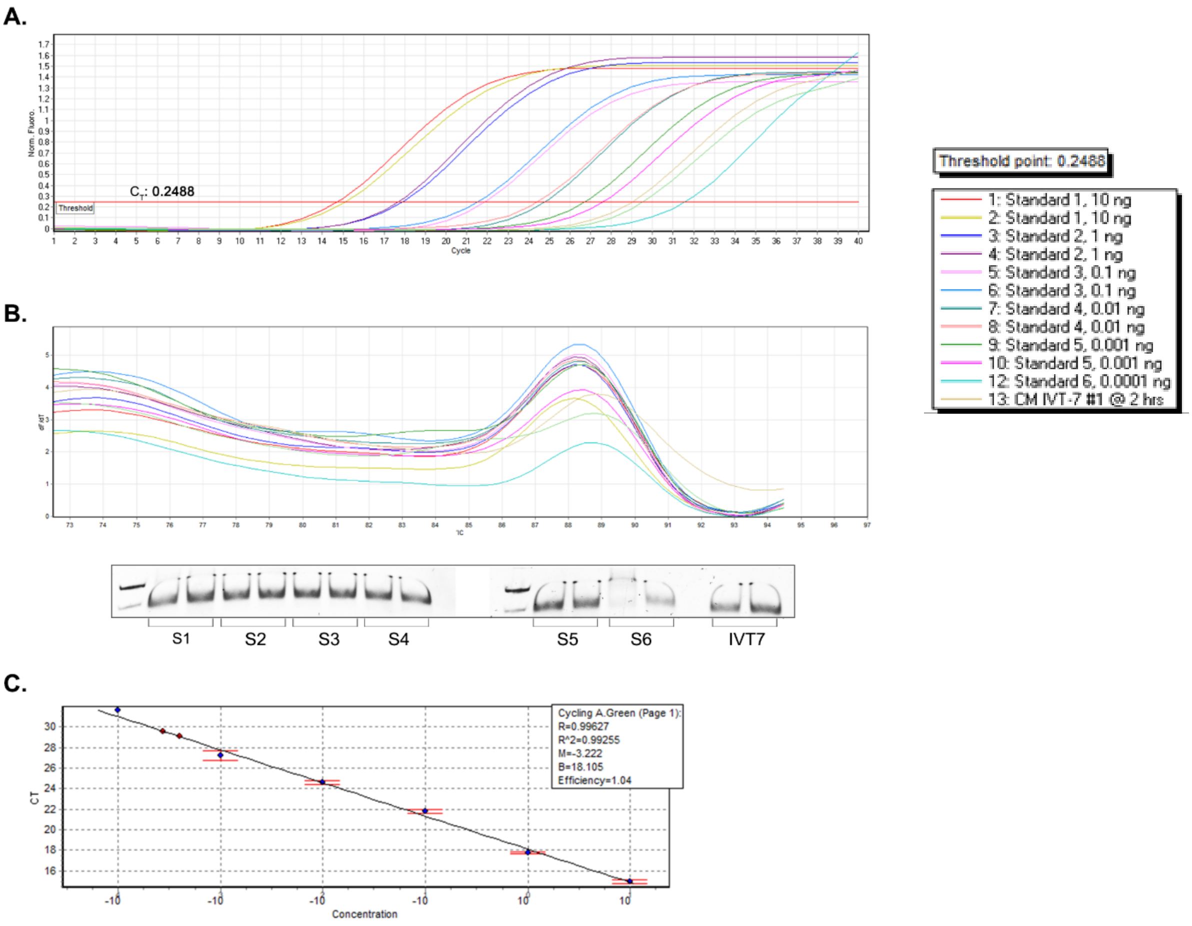

Figure S2. Identification of the linear dynamic range of RT-qPCR for absolute determination of aptamer amount, Related to Figures 3 and 6. A. Ten-fold serial dilutions of the standard's cDNA. Decrease in cDNA concentration results in a shift of C_T values to higher $C_{Ts.}$ **B.** Melt curve analysis of the RT-qPCR product demonstrating the production of a single peak, indicating the specificity of the qPCR primers for the target. The melt curve is plotted as the first derivative of fluorescence signal (dF) over the temperature (dT), (dF/dT). **Bottom:** Native polyacrylamide gel electrophoresis confirmation of the product following RT-qPCR. S1-S6, the serial dilutions; IVT-7, a representative sample from the selection to assess its amplification; Ladder, Ultra low range DNA ladder. **C.** Standard curve generated by plotting the serial dilutions (10-fold: 10 – 0.0001 ng) against the C_T values where unknown samples were interpolated. Each dilution was tested in duplicates. All graphs were plotted using the Rotor-Gene Q software. The gel image in panel B. was subjected to cropping for purposes of clarity.

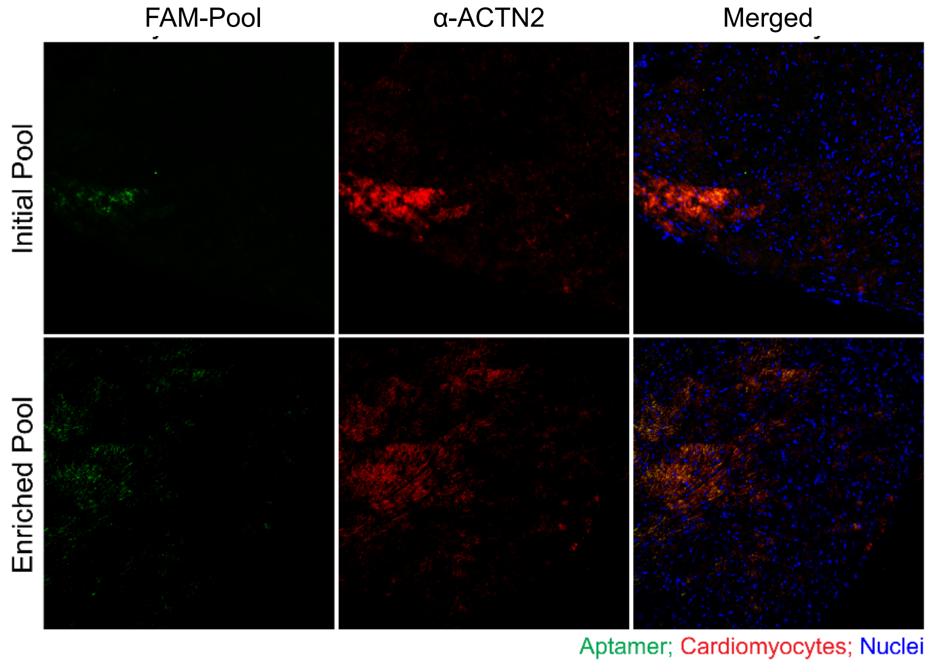

Figure S3. Aptamer pool localization in healthy, ventricular CMs, Related to Figure 3. IV injection in C57BL/10 mice (n= 3 per aptamer) of 2 nmol (~50 µg) FAM-labelled initial 2′F-Py RNA pool or enriched 2′F-Py RNA pool 7 (green) followed by collection of tissues 2h after injection. Sections were immunostained with α-ACTN2 to identify CMs (red) and counterstained with Hoechst 33342 nuclear stain (blue). Representative images of the left ventricle, where most CMs localize, were captured at the same laser intensities and acquisition time. Areas of colocalization are depicted in yellow (scale bar =20 um).

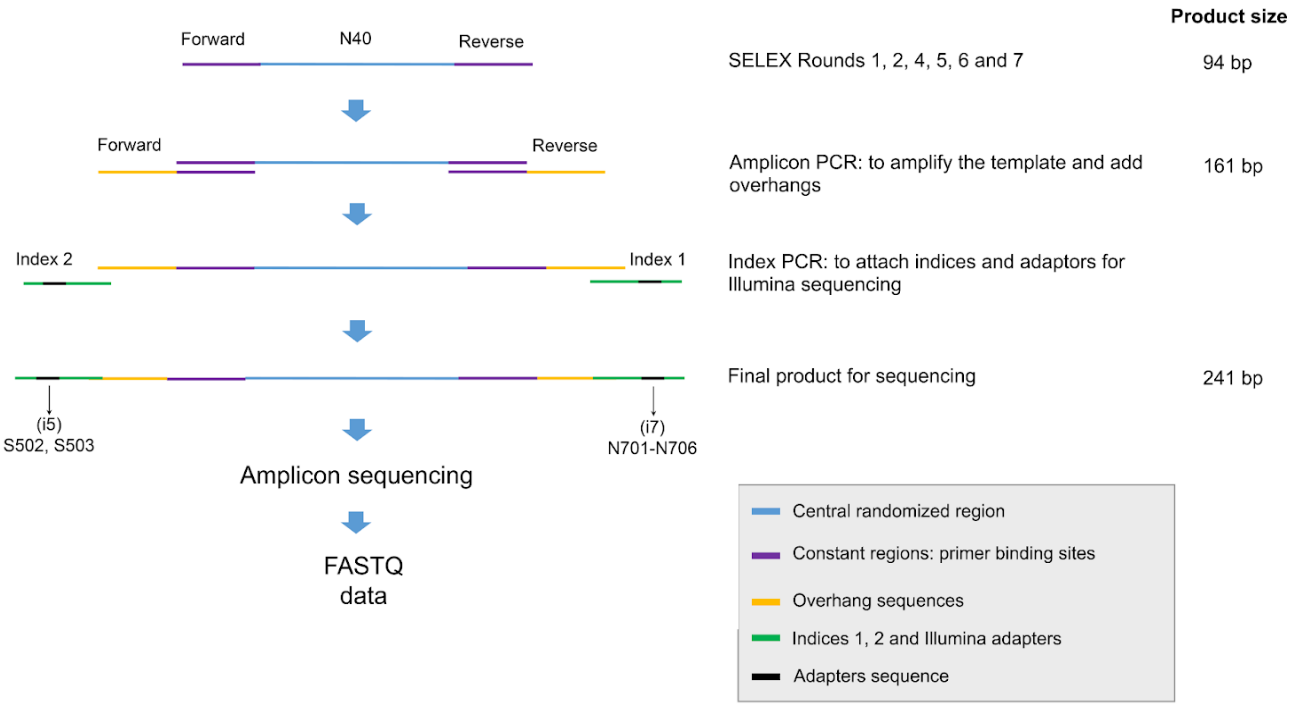

Figure S4. Aptamer libraries preparation for NGS analysis, Related to Figure 4. The diagram shows the steps required to prepare each aptamer library (recovered from CMs) for NGS using a modified 16S Metagenomics Library preparation protocol (Illumina). The aptamer library (top row) goes through two sequential PCR amplifications for the stepwise incorporation of the adapter overhangs (amplicon PCR) at first, and then the unique indices for each selection round (index PCR) using the primers shown in **Table S1**.

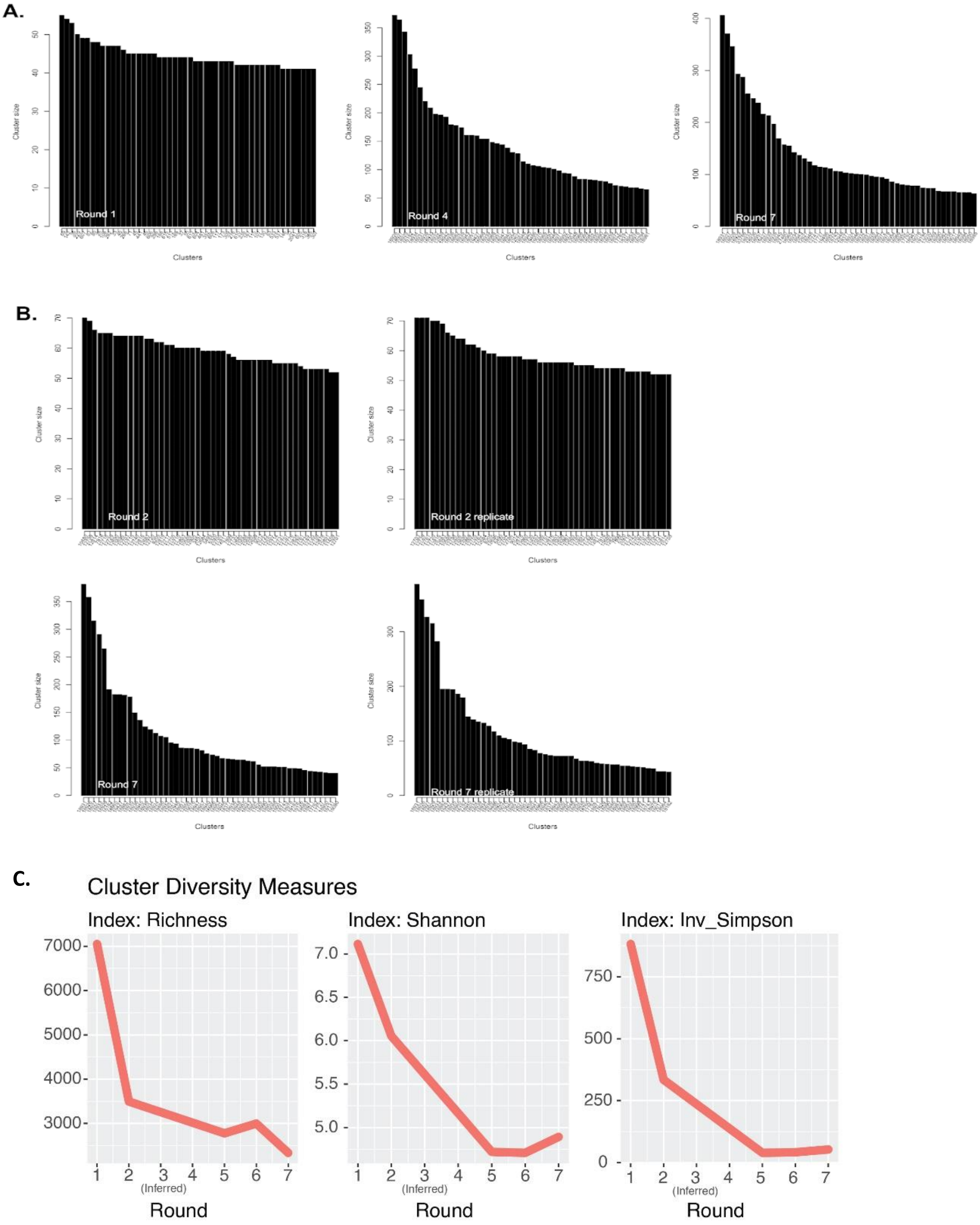

Figure S5: Enrichment of CM-enriched aptamers via NGS, Related to Figure 4 and 5. Distribution of sequences in each round and categorization into clusters according to sequence similarity. Histograms were produced from Illumina sequencing data. **A. and B.** The black bars show the number of member sequences (y-axis) in each cluster (x-axis). **A.** Histograms from selected rounds (Round 1, 4 and 7) demonstrating pool enrichment. Pool

enrichment is indicated by the change in cluster size distribution with the shift from approximately uniform to a skewed distribution. **B.** Cluster histograms of technical replicates (Round 2 and 7) demonstrate data reproducibility during library preparation for NGS analysis. **C.** Cluster diversity indices across SELEX rounds. Richness, Shannon and Inverse Simpson all indicate decreasing diversity as a function of SELEX rounds. The index unit, for all three panels, is a numerical value.

A.

```
11601        -----CGACGGACAAGGCUCACC----GUGGCCAUGUGAGCUCGGGCGC     40
10984        --AUGUCACGAACGAGGGCGUGCUCGCGUGGUGCGGAGGCA--------     39
18725        CGGAGCCACCGGCGCGUGGGUGCGGGUGCGGCCACCAGCA---------     40
10478        ---UG-CAGGUGCAUGUGGGAUCACGCGCGGUUAGGUCGCCGCG-----     40
10476        ---UGCGACGUGGGCGCGUCAUGCUGCGCGGUGCUGUGCACGC------     40
                    *       *           * **
```

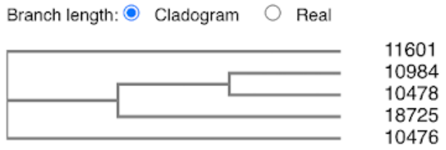

B.

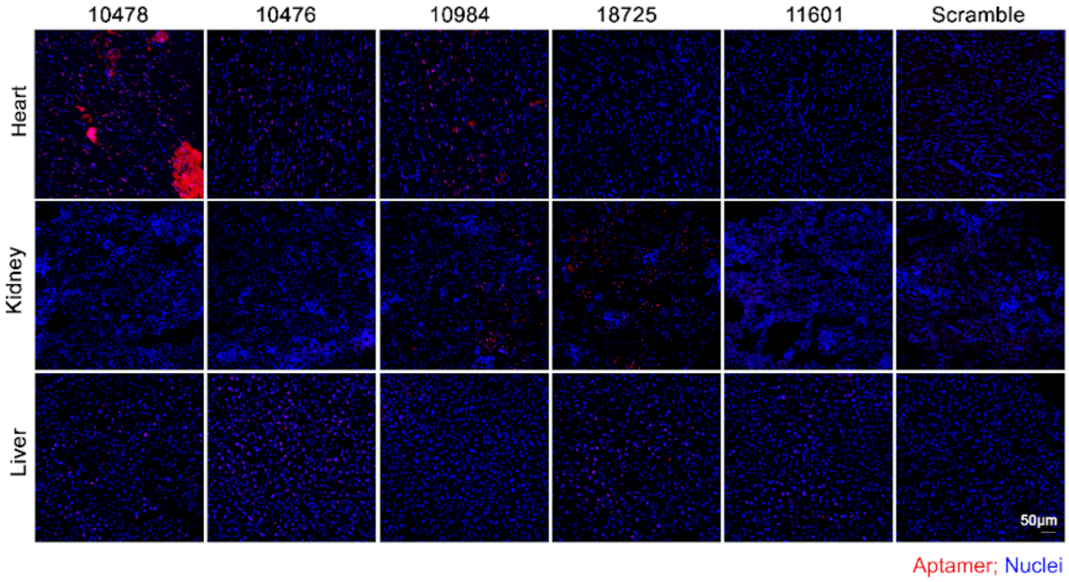

Figure S6. Assessment of additional aptamer candidates, Related to Figures 5 and 6. A. The consensus sequences (top) of the random region of selected 2′F-Py RNA aptamers aligned using Clustal Omega to reveal possible sequence homology (asterisks). Phylogenetic tree (bottom) representing the evolutionary relationship among the selected sequences. **B.** Cryosections from selected organs of *mdx* mice (n=2 per aptamer) incubated with 200 nM Alexa Fluor 647-labelled 2′F-Py RNA aptamers or Scramble control and counterstained with Hoechst 33342 nuclear stain (blue) (scale bar = 50 μm). White arrowheads in 10478 denote artefacts and/or debris and not specific signal. Punctate staining (red) denotes bound aptamer molecules.

2′F-Py RNA aptamer	MFE structure	Helicity	MFE (kcal/mol)
10478	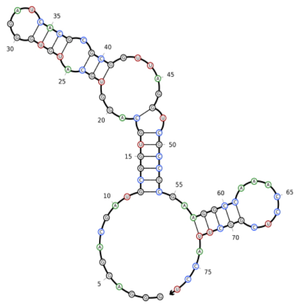	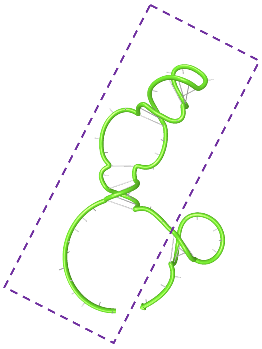	-22.60
10476	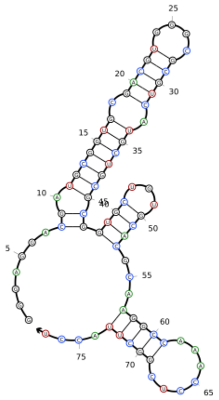	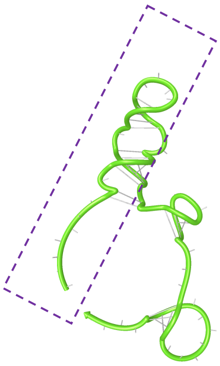	-26.50
10984	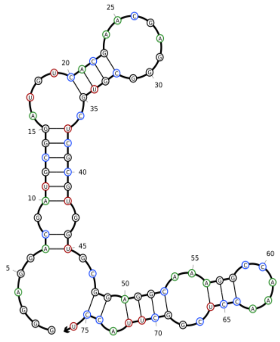	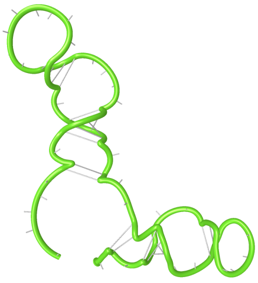	-19.70

**1**<br>**8**<br>**7**<br>**2**<br>**5**	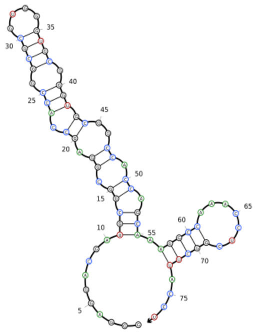	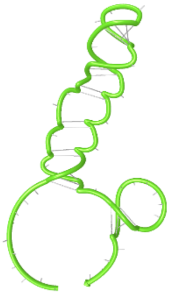	-24.50
**1**<br>**1**<br>**6**<br>**0**<br>**1**	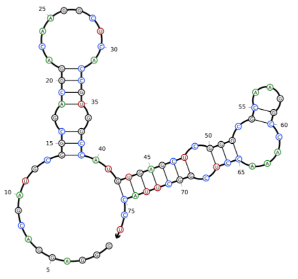	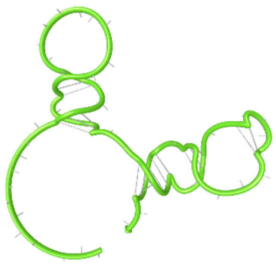	-22.20

Figure S7. Predicted structures, Related to Figures 5 and 6. Secondary structure predictions of selected 2'F-Py RNA aptamers 10478, 10476, 10984, 18725 and 11601. For each aptamer, the secondary structure (left) with the lowest (minimum) free energy (MFE) was determined using NUPACK at default settings. The helical depiction of each structure is shown on the right. Purple rectangle denotes similarities in loops and stems between the two helical depictions.

A.

Round #	Library concentration	# mice injected	Library circulation time	Initial aptamer detection cycle
1	3 nmol	3	2 hrs	30
2	5 nmol	3	2 hrs	28
3	5 nmol	3	2 hrs	26
4	5 nmol	3	2 hrs	25
5	5 nmol	3	2 hrs	25
6	5 nmol	3	2 hrs	22
7	5 nmol	3	2 hrs	22
8	5 nmol	3	2 hrs	22
9	5 nmol	3	2 hrs	22

B.

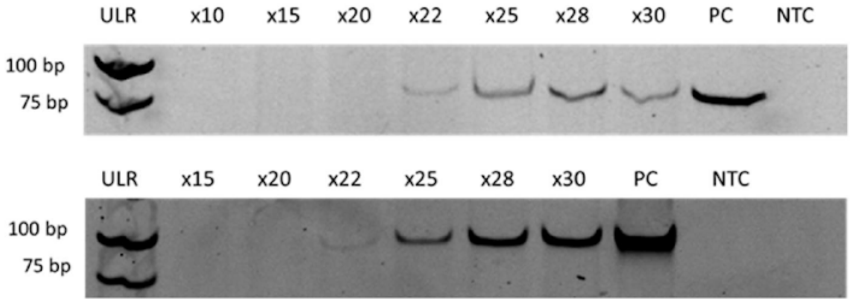

Figure S8: Confirmatory rounds *In vivo* SELEX conditions and indirect monitoring of aptamer recovery via PCR, Related to Figures 4 and 5. A. The rectangle denotes the extra 2 selection rounds performed to confirm pool convergence. **B.** Native polyacrylamide gel confirmation of the successful recovery of aptamer sequences (94 bp PCR product size) at cycle 22 from cardiomyocytes, following the confirmatory rounds 8 and 9 (n=3 adult hearts/round). Recovery was assessed via conventional PCR utilizing primers specific for the starting library. NTC: No template control; PC: Positive control; ULR: Ultra low range DNA ladder; ssRNA: Low range ssRNA ladder; x15 - x30: PCR cycles. Gel images were subjected to cropping for purposes of clarity.

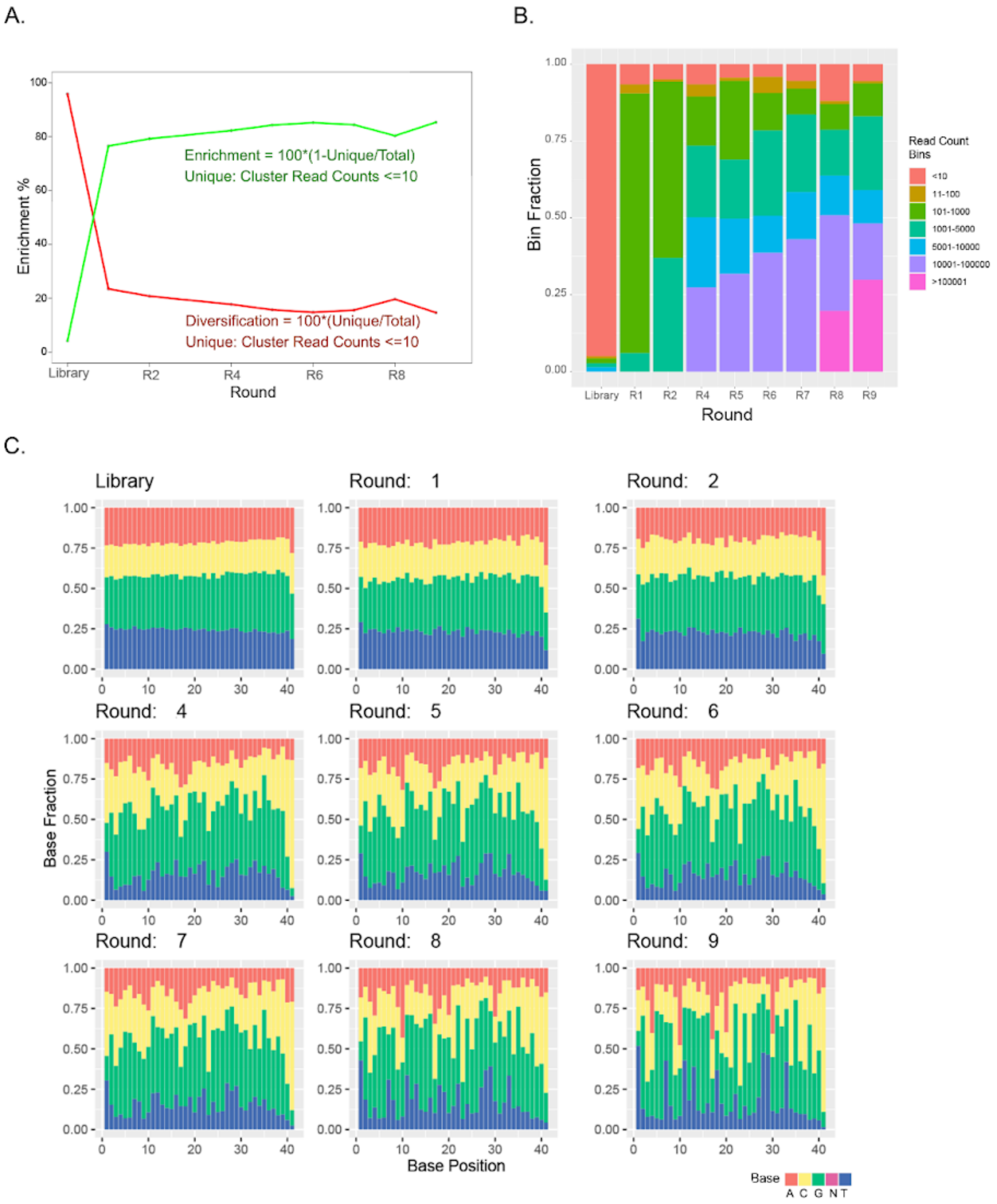

Figure S9: Confirmation of cardiomyocyte-targeted 2'F-Py RNA aptamers enrichment, Related to Figures 4 and 5. NGS analysis of the aptamer pools was repeated. The NGS run included Rounds 1-7 as well as the initial library (i.e., round 0) and the confirmatory Rounds 8 and 9, to confirm the enrichment plateau. **A.** Analysis of unique sequences in the initial pool and enriched pools 1-9. Percent of enrichment (%) and diversification in each pool were used as measures of the levels of enrichment and presence of unique sequences (enrichment and diversification were determined using the equations shown in the panel). **B.** Nucleotide distribution over the random region denoted as the frequency of each base per position over the total number of reads, where total number of reads equals 1. **C.** Data categorization into bins according to frequencies denoted as the proportion of each bin over the total population of reads on the dataset, where total reads population equals to 1.

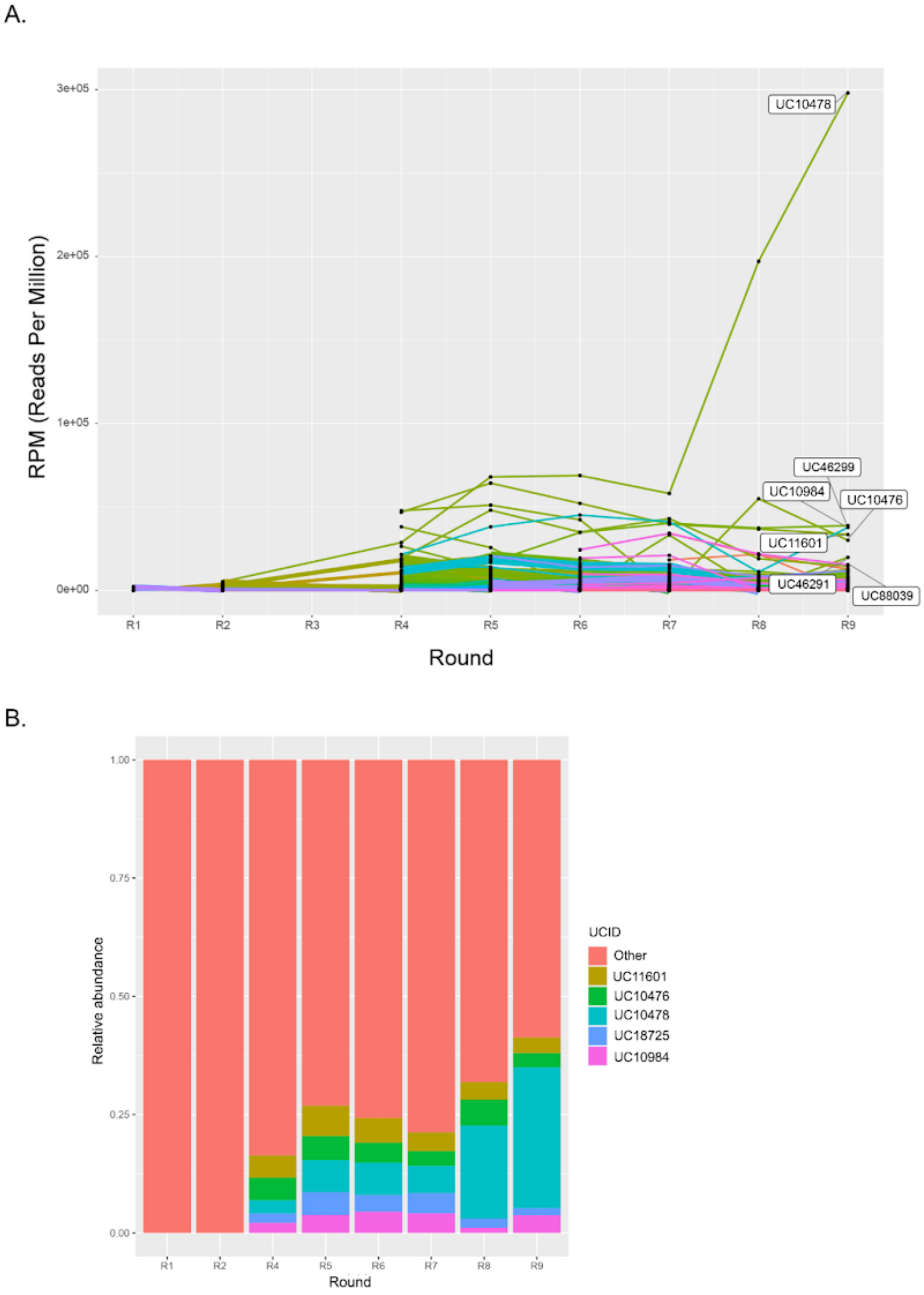

Figure S10: Aptamer cluster tracking across SELEX and frequencies of specific candidates, Related to Figures 4 and 5. A. Cluster matching across rounds. Aptamer sequences from each round were clustered in groups according to sequence similarities with their frequencies reported as RPM (Y-axis). Colours depict individual aptamer clusters that progress through rounds. The clusters with the highest number of reads (frequency) at the final SELEX round are denoted by a unique identification number (UC#). **B.** Relative abundance (frequency) for selected clusters UC10478, UC10476, UC10984, UC18725 and UC11601 across the selection. "Other" denotes other clusters and unique (random) sequences present at low frequencies

(≤50 counts). The relative abundance is denoted as the proportion of read counts of the selected clusters over the population of the total reads, where total reads =1.

Table S1. List of Oligonucleotide Sequences and Primer Pairs

Oligonucleotide	Sequence (5′-3′)	Length
**DNA library template (N40.2)**	TAATACGACTCACTATAGGGAGGACGATGCGGNNNNNNNNNNNNNNNNNNNNNNNNNNNNNNNNNNNNNNNNNNNNNNNAAGGCCAAACCTCGGCTTACCT	94 b
**2'F-Py RNA aptamer library (N40.2)**	GGGAGGACGAUGCGGNNNNNNNNNNNNNNNNNNNNNNNNNNNNNNNNNNNNNNNNAAGGCCAAACCUCGGCUUACCU	77 b
**2'F-Py RNA aptamer 10478**	GGGAGGACGAUGCGGUGCAGGUGCAUGUGGGAUCACGCGCGGUUAGGUCGCCGCGAAGGCCAAACCUCGGCUUACCU	77 b
**2'F-Py RNA aptamer 10476**	GGGAGGACGAUGCGGUGCGACGUGGGCGCGUCAUGCUGCGCGGUGCUGUGCACGCAAGGCCAAACCUCGGCUUACCU	77 b
**2'F-Py RNA aptamer 10984**	GGGAGGACGAUGCGGAUGUCACGAACGAGGGCGUGCUCGCGUGGUGCGGAGGCAAAGGCCAAACCUCGGCUUACCU	76 b
**2'F-Py RNA aptamer 18725**	GGGAGGACGAUGCGGCGGAGCCACCGGCGCGUGGGUGCGGGUGCGGCCACCAGCAAAGGCCAAACCUCGGCUUACCU	77 b
**2'F-Py RNA aptamer 11601**	GGGAGGACGAUGCGGCGACGGACAAGGCUCACCGUGGCCAUGUGAGCUCGGGCGCAAGGCCAAACCUCGGCUUACCU	77 b
**2'F-Py Scramble RNA control**	GGGCCGGUCGUGCCGCAAACCUCGUAGGUCGGCUAAUAAUGGCAUUAACGUCGAUUGGCACCAACCGAUUCCGCCAU	77 b
**SELEX Forward primer (SXFP1,2)**	TAATACGACTCACTATAGGGAGGACGATGCGG	32 b
**SELEX Reverse primer (SXRP2,3)**	AGGTAAGCCGAGGTTTGGCCTT	23 b
**18S rRNA Forward (mouse)**	TTGACGGAAGGGCACCACCAG	21 b
**18S rRNA Reverse (mouse)**	GCACCACCACCCACGGAATCG	21 b

**RT-qPCR SELEX Forward (set 1)**	TCACTATAGGGAGGACGATGCGG	23 b
**RT-qPCR SELEX Reverse (set 1)**	AGGTAAGCCGAGGTTTGGCCTT	22 b
**RT-qPCR SELEX Forward (set 2)**	GGGAGGACGATGCGG	15 b
**RT-qPCR SELEX Reverse (set 2)**	CCGAGGTTTGGCCTT	15 b
**NGS Forward primer**	**TCGTCGGCAGCGTCAGATGTGTATAAGAGACAG**T AATACGACTCACTATAGGGAGGACGATGCGG	65 b
**NGS Reverse primer**	**GTCTCGTGGGCTCGGAGATGTGTATAAGAGACAG** AGGTAAGCCGAGGTTTGGCCTT	56 b
**Nextera XT Index 1 Read** (for NGS reverse primer)	CAAGCAGAAGACGGCATACGAGAT- *[i7]* -GTCTCGTGGGCTCGG	47 b
**Nextera XT Index 2 Read** (for NGS forward primer)	AATGATACGGCGACCACCGAGATCTACAC- *[i5]* - TCGTCGGCAGCGTC	51 b
T7 promoter sequence is underlined; N denotes mixed base with 25% of each base (A, G, C, T); the random region sequence is highlighted in selected aptamer sequences; NGS forward and reverse overhang adapter sequence are in bold letters; Adapter indices (unique for each SELEX round), [i7] and [i5], are *italicised*.		

**Table S2.** Nextera XT adapter combinations for NGS samples		
**Sample**	**Index 1 (i7)**[a]	**Index 2 (i5**[a]
Initial Library	N701	S503
Round 1	N701	S502
Round 2	N702	S502
Round 4	N703	S502
Round 5	N704	S502
Round 6	N705	S502
Round 7	N706	S502
Round 8	N702	S503
Round 9	N703	S503
Round 10	N704	S503

[a]Adapter sequences can be found in the 16S Metagenomics Sequencing Library Preparation protocol: Dual Indexing Principle.

**Table S3. Frequency of selected clusters in Round 7**		
**Ranking**	**Cluster**	**Frequency in RPM**
1	UC10478	62000
2	UC10476	47500
3	UC18725	44167
4	UC10984	43334
5	UC11601	41667

**Table S4.** Average body weight and organ weights in *C57BL/10ScSn-Dmd^{mdx}/J*.		
**Body Weight (grams)[a]**	**Age**	**Gender**
24.89 g ± 0.1847	6-8 weeks	Male
**Organ Harvested**	**Organ weight (grams)[a]**	**% Body weight[a]**
Heart	0.2524 ± 0.02	1.014 ± 0.077
CM fraction[b] of the heart	0.0508 ± 0.015	0.204 ± 0.062
Gastrocnemius (left)	0.1520 ± 0.0014	0.6108 ± 0.0058
Quadriceps (left)	0.1923 ± 0.0218	0.773 ± 0.0877
Liver	1.5777 ± 0.109	6.339 ± 0.4391
Kidney (left)	0.1648 ± 0.0139	0.6622 ± 0.056
Lungs	0.2158 ± 0.048	0.8671 ± 0.1927

[a]Values are presented as mean ± SD, n=3.
[b]The weight of the CM fraction was calculated as follows:
(weight of the Falcon tube with the CMs pellet [c]) - (weight of empty Falcon tube before CM isolation).
[c]CMs pellet is defined as the CMs following the final gravity settling round and complete removal of the perfusion buffer from the tube.

Statistical Analysis: The data presented in graphs 3A and 5C are expressed as fold change (FC) of the initial pool (T0=1). As the fold change is not normally distributed, the data were first long-transformed (log_2FC) and then tested whether the average of the enriched pool 7 (T7) or aptamer 10478 differs significantly from 0 [$log_2(1)=0$].

**Figure 3A**	**FC**	**Log2FC**
**Initial pool**	1	0
**Enriched pool 7 (mean)**	3.22	1.687
**SD**	1.544	0.6267
**n**	3	3
**Two-tailed P value**		0.0431*

*By conventional criteria, this difference is considered to be statistically significant.

**Figure 6C**	**FC**	**Log2FC**
**Initial pool**	1	0
**Aptamer 10478**	13.21	3.788
**SD**	6.565	2.715

**n**	3	3
**Two-tailed P value**		0.1369
*By conventional criteria, this difference is considered to be not statistically significant.		
